# Regulated GATA1 expression as a universal gene therapy for Diamond-Blackfan anemia

**DOI:** 10.1016/j.stem.2024.10.012

**Published:** 2024-11-11

**Authors:** Richard A. Voit, Xiaotian Liao, Alexis Caulier, Mateusz Antoszewski, Blake Cohen, Myriam Armant, Henry Y. Lu, Travis J. Fleming, Elena Kamal, Lara Wahlster, Aoife M. Roche, John K. Everett, Angelina Petrichenko, Mei-Mei Huang, William Clarke, Kasiani C. Myers, Craig Forester, Antonio Perez-Atayde, Frederic D. Bushman, Danilo Pellin, Akiko Shimamura, David A. Williams, Vijay G. Sankaran

**Affiliations:** 1 Division of Hematology/Oncology, Boston Children’s Hospital, Harvard Medical School, Boston, MA 02115, USA; 2 Department of Pediatric Oncology, Dana-Farber Cancer Institute, Harvard Medical School, Boston, MA 02215, USA; 3 Howard Hughes Medical Institute, Boston, MA 02115, USA; 4 Broad Institute of MIT and Harvard, Cambridge, MA 02142, USA; 5 Boston Children’s Hospital, Harvard Medical School, Boston, MA 02115, USA; 6 Department of Microbiology, University of Pennsylvania, Perelman School of Medicine, Philadelphia, PA 19104, USA; 7 Division of Bone Marrow Transplantation and Immune Deficiency, Cincinnati Children’s Hospital Medical Center, Cincinnati, OH 45229, USA; 8 University of Cincinnati College of Medicine, Cincinnati, OH 45267, USA; 9 Division of Pediatric Hematology/Oncology/Bone Marrow Transplant, Children’s Hospital Colorado, University of Colorado Anschutz Medical Campus, Aurora, CO 80045, USA; 10 Present Address: Department of Pediatrics, University of Texas Southwestern Medical Center, Dallas, TX 75390, USA; 11 Lead Contact

## Abstract

Gene therapy using hematopoietic stem and progenitor cells is altering the therapeutic landscape for patients with hematologic, immunologic, and metabolic disorders but has not yet been successfully developed for individuals with the bone marrow failure syndrome Diamond-Blackfan anemia (DBA). More than 30 mutations cause DBA through impaired ribosome function and lead to inefficient translation of the erythroid master regulator GATA1, providing a potential avenue for therapeutic intervention applicable to all patients with DBA, irrespective of the underlying genotype. Here, we report the development of a clinical-grade lentiviral gene therapy that achieves erythroid-lineage restricted expression of GATA1. We show that this vector is capable of augmenting erythropoiesis in DBA models and diverse patient samples, without impacting hematopoietic stem cell function or demonstrating any signs of premalignant clonal expansion. These preclinical safety and efficacy data provide strong support for the first-in-human universal gene therapy trial for Diamond-Blackfan anemia through regulated GATA1 expression.

## eTOC

Gene therapy for Diamond-Blackfan anemia is constrained by dozens of causative mutations. Voit and colleagues develop a universal gene therapy through erythroid lineage restricted expression of GATA1 that rescues the erythroid impairment of DBA without impacting HSC function. These data provide support for the first-in-human universal gene therapy trial for DBA.

## Graphical Abstract

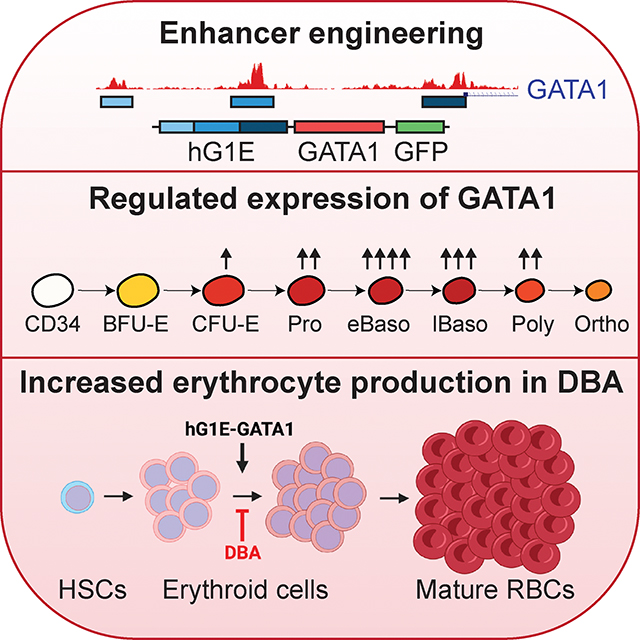

## Introduction

The success of gene therapies targeting hematopoietic stem and progenitor cells (HSPCs) has established novel treatment paradigms for patients with hemoglobin disorders, immunodeficiencies, and metabolic diseases^1–3^. Nonetheless, gene therapy for inherited bone marrow failure syndromes like Diamond-Blackfan anemia (DBA) has been limited by heterogeneous causative mutations and small patient numbers^4^. DBA is characterized by severely impaired red blood cell production, and the only treatment options are chronic transfusions, corticosteroids, or hematopoietic stem cell transplantation. More effective, safer, and permanent cures for DBA are lacking, but few drugs are in active development, and suitable donors do not exist for all patients. Racial and ethnic disparities in allogeneic bone marrow transplantation exacerbate this unmet need for DBA patients from minority groups^5^.

Mutations in up to 37 genes have been implicated in DBA or DBA-like syndromes^6^. Monoallelic mutations in the ribosomal protein gene *RPS19* are present in approximately 25% of patients with DBA, and mutations in other ribosomal protein or related genes account for most of the remaining known genotypes^6–8^. *RPS19* gene addition by lentiviral delivery^9–11^ has demonstrated preclinical success but is not applicable to the >75% of DBA patients without *RPS19* mutations. DBA mutations converge to impair the level of ribosomes, which preferentially alters the translation of a subset of mRNAs, most notably the hematopoietic master regulator *GATA1*^12^. In addition, loss-of-function *GATA1* mutations have been identified which themselves can cause DBA^13^.

We previously showed that increasing the expression of GATA1 in bone marrow samples from patients with DBA is sufficient to overcome the erythroid differentiation defects *in vitro^14^*, suggesting that restoration of sufficient GATA1 protein levels could be a therapeutic avenue for DBA patients regardless of the underlying genotype. However, unregulated GATA1 expression in HSCs promotes premature erythroid differentiation at the expense of long-term HSC maintenance^15,16^, necessitating a strategy of lineage-specific augmentation of GATA1 expression.

Here, we identify endogenous regulatory elements which enable lineage-restricted and highly regulated expression of GATA1 in human hematopoietic cells. We have engineered a lentiviral gene therapy vector that integrates into long-term (LT-) HSCs but only drives exogenous GATA1 expression once progenitors commit to erythroid differentiation. We demonstrate that regulated GATA1 expression is sufficient to overcome the erythroid maturation arrest in models of DBA and in primary DBA patient samples, without perturbing HSC function. These data will enable clinical trials of the first-in-human, universal gene therapy for Diamond-Blackfan anemia. Importantly, this work extends the reach of HSC-based gene therapies, demonstrating for the first time how regulated expression of a single transgene in a clinically applicable manner leads to functional correction of the hematopoietic defects arising due to mutations in dozens of genes.

## Results

### Construction of an erythroid-specific enhancer

**Figure 1.**
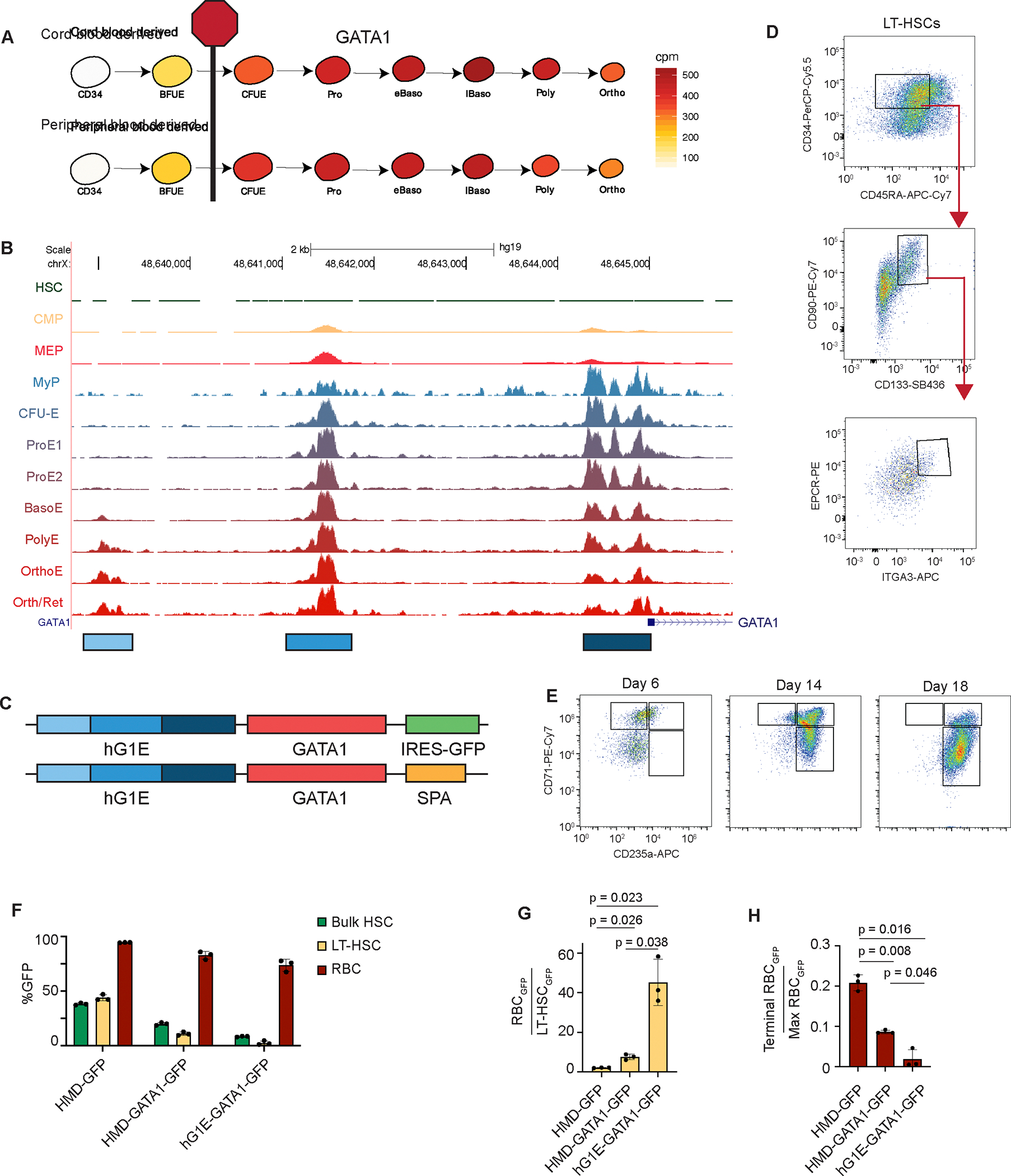
Endogenous enhancer elements drive erythroid-restricted transgene expression. **A.** GATA1 mRNA expression (cpm) during normal human erythroid differentiation from umbilical cord blood or peripheral mobilized CD34-selected HSPCs^17^. Stage of erythroid maturation arrest in DBA is shown. **B.** Accessible chromatin upstream of the GATA1 transcriptional start site in HSCs^20^ and progenitors undergoing erythroid differentiation^21^. Peak height is scaled to the highest peak and the displayed range is from 0–1.8 in each population. The three differentially accessible regions marked were used to construct the hG1E element. **C.** Lentiviral constructs designed to achieve erythroid-restricted expression. IRES – internal ribosome entry site, GFP – green fluorescent protein, SPA – synthetic polyA. **D.** FACS gating strategy to enrich for LT-HSCs on day 6 of HSC culture. LT-HSCs are defined as CD34^+^ CD45RA^−^ CD90^+^ CD133^+^ EPCR^+^ ITGA3^+^. **E.** Representative FACS plots from the three phase *in vitro* erythroid differentiation of transduced human HSPCs. Differentiation was assessed by expression of CD71 and CD235a by flow cytometry at the indicated days of erythroid culture. **F.** Differential transgene expression in HSCs and erythroid progenitors. The percentage of GFP positive cells from the bulk population (bulk HSCs) or the LT-HSC population on day 6 of HSC culture, or from the CD71^+^CD235a^+^ population (RBC) on day 6 of erythroid culture was determined by flow cytometry. n = 3 independent replicates, mean and S.E.M. are shown. **G.** Ratio of GFP expression in erythroid progenitors compared to LT-HSCs. Percentage of cells expressing GFP was compared between the CD71^+^CD235a^+^ population on day 6 of erythroid culture and the LT-HSC population on day 6 of HSC culture. n = 3 independent replicates, mean and S.E.M. are shown. Two-sided Student *t*-test was used for comparisons, and P values are shown. **H.** Ratio of GFP expression during terminal differentiation. Percentage of cells expressing GFP was compared between the CD71^−^CD235a^+^ population on day 18 of erythroid culture and CD71^+^CD235a^+^ erythroid progenitors on day 6. n = 3 independent replicates, mean and S.E.M. are shown. Two-sided Student *t*-test was used for comparisons, and P values are shown. See also Figure S1.

To enable a universally applicable gene therapy for DBA, we sought to modify engraftable LT-HSCs with a *GATA1* transgene that is only expressed after erythroid lineage commitment, and that mimics the temporal expression pattern of endogenous *GATA1*^17^ (Fig. 1A). Motivated by prior work in mouse hematopoiesis^18,19^, we examined accessible chromatin upstream of the *GATA1* transcriptional start site in human HSCs^20^ and cells undergoing erythroid differentiation^21^ to identify human regulatory enhancers that enable erythroid lineage-restricted *GATA1* expression. We identified three regions of DNA that are accessible exclusively during erythroid maturation (Fig. 1B) and are bound by erythroid transcription factors (Fig. S1A). To validate these regions as lineage-restricted enhancers, we concatenated them to generate the human GATA1 enhancer (hG1E) element and used it to drive expression of codon-optimized *GATA1* cDNA to maximize GATA1 protein synthesis in erythroid progenitors. Our initial version also expressed GFP from the same hG1E sequence linked to the *GATA1* cDNA by an internal ribosomal entry site (IRES) sequence (Fig. 1C), and a subsequent version was a clinical-grade lentivirus with a synthetic polyadenylation sequence (SPA)^22,23^ in a backbone previously utilized in human clinical studies^24^.

We tested expression of the hG1E-GATA1-IRES-GFP cassette, in comparison with constitutively expressing HMD-GFP and HMD-GATA1-IRES-GFP, in primary human CD34^+^ HSPCs collected from healthy human adult donors or umbilical cord blood. We split the transduced cultures into HSC media^25^ (Fig. 1D) and erythroid differentiation media^26^ (Fig. 1E). We observed 5-fold lower expression of GFP from the regulated hG1E vector in the bulk population of HSPCs, and almost 20-fold lower expression in the immunophenotypic LT-HSC population compared to controls (Fig. 1F). We found no difference in the proportion of LT-HSCs expressing GATA1 protein following hG1E-GATA1 treatment, but observed a significantly higher percentage of GATA1^+^ LT-HSCs in the HMD-GATA1 cohort (Fig. S1B). This constitutive GATA1 expression from the HMD-GATA1 vector led to a decrease in LT-HSC maintenance, consistent with previous observations^15^, but regulated GATA1 expression from our hG1E cassette did not impact LT-HSC numbers (Fig. S1C).

In contrast, there was transgene expression in more than 75% of developing erythroid progenitors following hG1E-GATA1-IRES-GFP treatment (Fig. 1F). The expression pattern of GFP from the hG1E cassette during erythropoiesis (Fig. S1D) followed the expected temporal pattern of GATA1 expression (Fig. 1A). The ratio of GFP expression in CD71^+^CD235a^+^ erythroid progenitors compared to LT-HSCs was more than 40-fold higher in the hG1E-GATA1-IRES-GFP samples (Fig. 1G). Upon terminal erythroid differentiation when endogenous GATA1 levels decline, GFP expression from the hG1E cassette also decreased (Fig. 1H).

In parallel, we tested regulated expression from additional vectors, including shorter versions of hG1E, and those with binding sites for microRNAs that restrict expression in HSCs^27^ (Fig. S1E). We confirmed that expression from the parental hG1E cassette provides the most faithful restriction in HSCs with robust expression in developing erythroid cells (Fig. S1F). Collectively, these data reveal that the engineered hG1E regulatory element achieves erythroid lineage-restricted expression of transgenes that mimics the expression pattern of GATA1 during erythropoiesis.

### Exogenous regulated GATA1 expression does not impair erythroid differentiation and preserves HSC function

**Figure 2.**
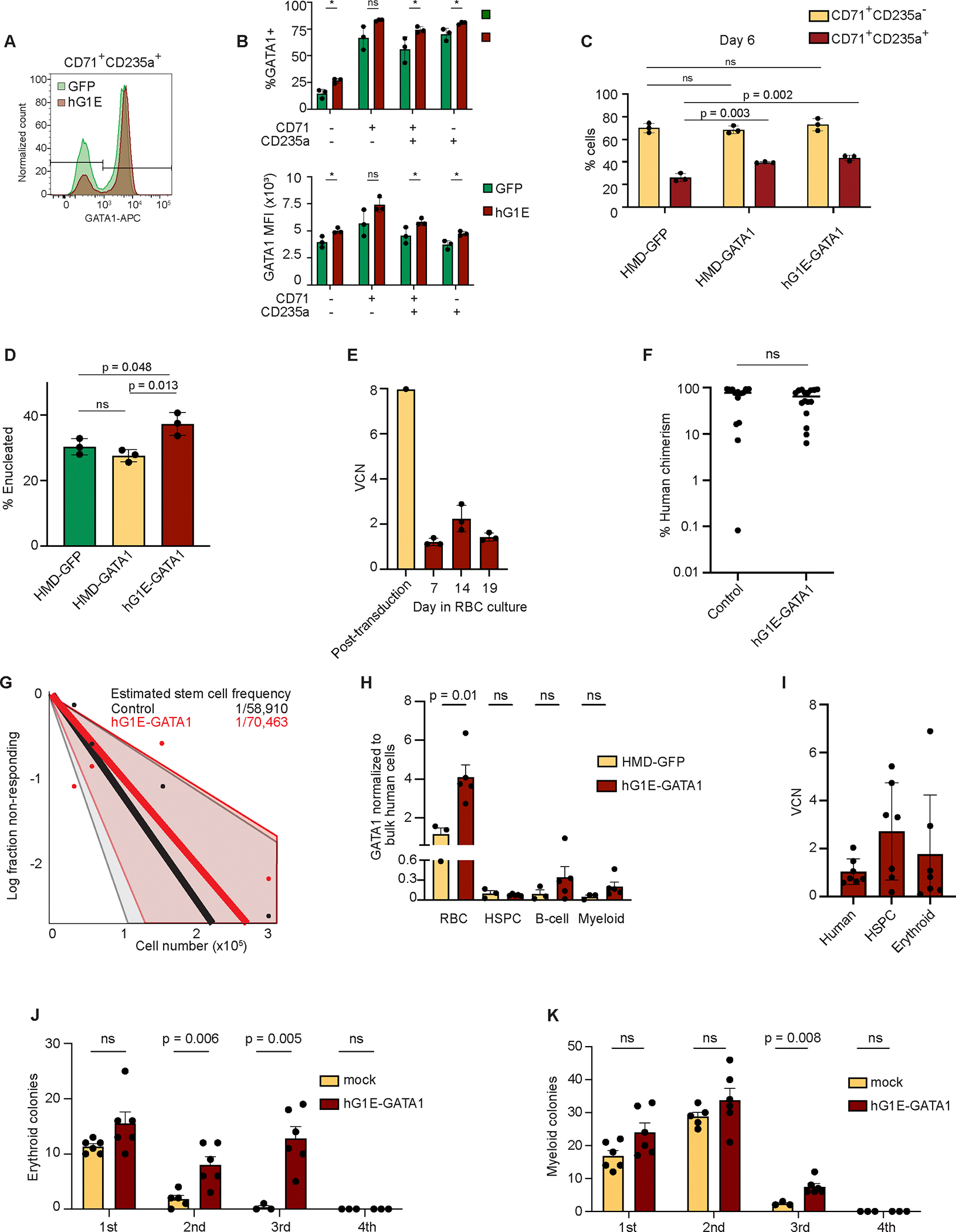
Regulated GATA1 expression preserves erythroid maturation and HSC function. **A.** Intracellular GATA1 protein level in single erythroid progenitors. On day 9 of erythroid culture, cells were fixed, permeabilized, and stained with antibodies against CD71 and CD235 as well as GATA1 and analyzed by flow cytometry. Level of GATA1 protein per cell is proportional to the fluorescence intensity displayed along the x-axis. **B.** Intracellular GATA1 expression following hG1E-GATA1 treatment. In each indicated erythroid population on day 9, the percentage of cells that express GATA1 (top) and the mean fluorescence intensity of GATA1 (bottom) are displayed. n = 3 independent replicates, mean and S.E.M. are shown. Two-sided Student *t*-test was used for comparisons. * P <0.05, ns - not significant. **C.** Erythroid differentiation after hG1E-GATA1-IRES-GFP treatment. On day 6 of erythroid culture, the stage of differentiation was assessed by flow cytometry. The percentage of CD71^+^CD235a^−^ cells and CD71^+^CD235a^+^ cells are shown. n = 3 independent replicates, mean and S.E.M. are shown. Two-sided Student *t*-test was used for comparisons. P values are shown. ns - not significant. **D.** Percentage of enucleated cells on day 21 of erythroid culture. Percentage of CD71^−^ CD235a^+^ cells that exclude Hoechst dye is displayed. n = 3 independent replicates, mean and S.E.M. are shown. Two-sided Student *t*-test was used for comparisons. P values are shown. ns - not significant. **E.** Preservation of transduced cells during *in vitro* erythroid culture. Vector copy number (VCN) analysis was performed following hG1E-GATA1 treatment on day 7 of HSC culture and on the indicated days in erythroid culture. n = 3 independent replicates of erythroid culture from the same pool of transduced cells. Mean and S.E.M. are shown. **F.** Effect of hG1E-GATA1 treatment on engraftment. 200,000 cells were transplanted into NBSGW mice after transduction with the indicated vector. Human chimerism was determined by flow cytometry of recipient bone marrow samples by comparing the number of cells expressing human or mouse CD45. Two-sided Student *t*-test was used for comparisons. ns - not significant. **G.** Extreme limiting dilution plot with estimated stem cell frequency. 25,000–350,000 human HSPCs were transplanted per recipient mouse after transduction. Data were generated over three independent experiments using the hG1E-GATA1 vector compared to either mock treated or HMD-GFP treated cells. Successful engraftment was defined as human chimerism of at least 0.1%. Estimated stem cell frequency was calculated using ELDA^49^. **H.** Lineage-restricted GATA1 expression *in vivo*. Bone marrow was collected from primary xenotransplant recipients and sorted for bulk human cells (CD45^+^), erythroid progenitors (CD71^+^), HSPCs (CD34^+^), B-cells (CD19^+^), and myeloid cells (CD33^+^). GATA1 expression normalized to bulk human cells of the same recipient is shown. n = 3–5 xenotransplant recipients as shown, mean and S.E.M. are shown. Two-sided Student *t*-test was used for comparisons. P values are shown. ns - not significant. **I.** Preservation of transduced cells *in vivo*. Cells from the transduction in Fig. 2E were transplanted into NBSGW mice and bone marrow samples were harvested at 16 weeks. VCN analysis was performed in human cell subpopulations, defined by the following human lineage markers: Human, CD45^+^; HSPC, CD34^+^, Erythroid, CD71^+^. **J. - K.** Quantification of colonies in serial replating assay after xenotransplantation. Human cells harvested from xenotransplant recipients were used for serial CFU quantification. In each round, 5e4 cells were plated in methylcellulose. BFU-E colony number (**J.**) and CFU-G, CFU-M, and CFU-GM colony number (**K.**) were quantified after 9 days using StemVision with manual verification. n = 3–6 independent replicates as shown by number of markers. Mean and S.E.M. are shown. Two-sided Student *t*-test was used for comparisons. P values are shown. ns - not significant. See also Figure S2.

We next assessed the impact of regulated GATA1 expression during erythropoiesis. Treatment with hG1E-GATA1-IRES-GFP increased the number of cells expressing GATA1 protein and the amount of GATA1 per cell, while preserving the temporal expression pattern in committed erythroid progenitors (Fig. 2A, B). Exogenous GATA1 expression resulted in a modest acceleration of early erythroid differentiation with a higher proportion of CD71^+^CD235a^+^ cells on day 6 compared to controls (Fig. 2C). By day 14, hG1E-GATA1-IRES-GFP samples had a normal distribution of erythroid precursor populations, but HMD-GATA1 cells showed continued skewing of erythroid differentiation with a higher proportion of cells in the CD71^+^CD235a^+^ population (Fig. S2A). Enucleation of mature RBCs was reduced in the HMD-GATA1 samples compared to the hG1E samples (Fig. 2D), suggesting that constitutive (but not regulated) GATA1 expression interferes with terminal erythroid differentiation as has been observed in mice^28^. Vector copy number (VCN) analysis revealed preservation of transduced cells (Fig. 2E).

Next, we sought to determine the functional effect of regulated GATA1 expression on HSC function *in vivo*. We treated human CD34^+^ HSPCs with the hG1E-GATA1 vector or controls followed by xenotransplantation into non-irradiated immunodeficient and Kit-mutant NOD.Cg-*Kit*^*W−41J*^*Tyr*^+^*Prkdc*^*scid*^*Il2rg*^*tm1Wjl*^/ThomJ (NBSGW) mice that allow for robust human hematopoiesis after xenotransplantation^25,29,30^. Recipient bone marrows revealed no difference in human cell engraftment in the hG1E treated samples (Fig. 2F). Upon limiting dilution xenotransplantation, we found no reduction in engraftable stem cell frequency in the hG1E-GATA1 treated group (Fig. 2G).

In parallel, we performed xenotransplantation of human HSPCs transduced with hG1E-GATA1-IRES-GFP. In the bone marrow, we observed no GFP expression from the hG1E cassette in human HSPCs, but rather only lineage-restricted expression in erythroid cells with minimal expression in myeloid cells (Fig. S2B), which might reflect endogenous GATA1 expression in eosinophils and basophils^31,32^. The hG1E-GATA1-IRES-GFP group showed increased GATA1 expression in human CD71^+^ erythroid cells, but not in human CD34^+^ HSPCs (Fig. 2H).

hG1E-GATA1 did not induce lineage-skewing *in vivo* in these healthy (non-DBA) human progenitors (Fig. S2C), suggesting a minimal impact on healthy human hematopoiesis. VCN analysis in total human cells, HSPCs, and erythroid progenitors revealed successful long-term engraftment of transduced cells (Fig. 2I). We found that erythroid progenitors with an average of 1.76 viral copies per genome resulted in ~3.5-fold higher expression of GATA1 in an erythroid-restricted manner.

Finally, we performed secondary xenotransplantation using human hematopoietic cells purified from primary recipients. We found no difference in the proportion of engrafted recipients or overall human chimerism (Fig. S2D), although the level of chimerism was low as expected for secondary transplants with adult peripheral blood mobilized HSPCs^33^, preventing the analysis of lineage output. Therefore, we performed serial CFU assays from bone marrow samples collected from primary recipients. We observed erythroid (Fig. 2J) and myeloid (Fig. 2K) colonies in three rounds of replating, but no colonies in the fourth round of replating consistent with a lack of aberrant clonal expansion. We observed significantly more erythroid colonies in the second and third rounds of replating in hG1E-GATA1 samples. Together, these data demonstrate that hG1E-GATA1 treatment supports increased erythropoiesis and preserves HSC function *in vivo*.

### hG1E-GATA1 treatment improves erythroid output in DBA models

**Figure 3.**
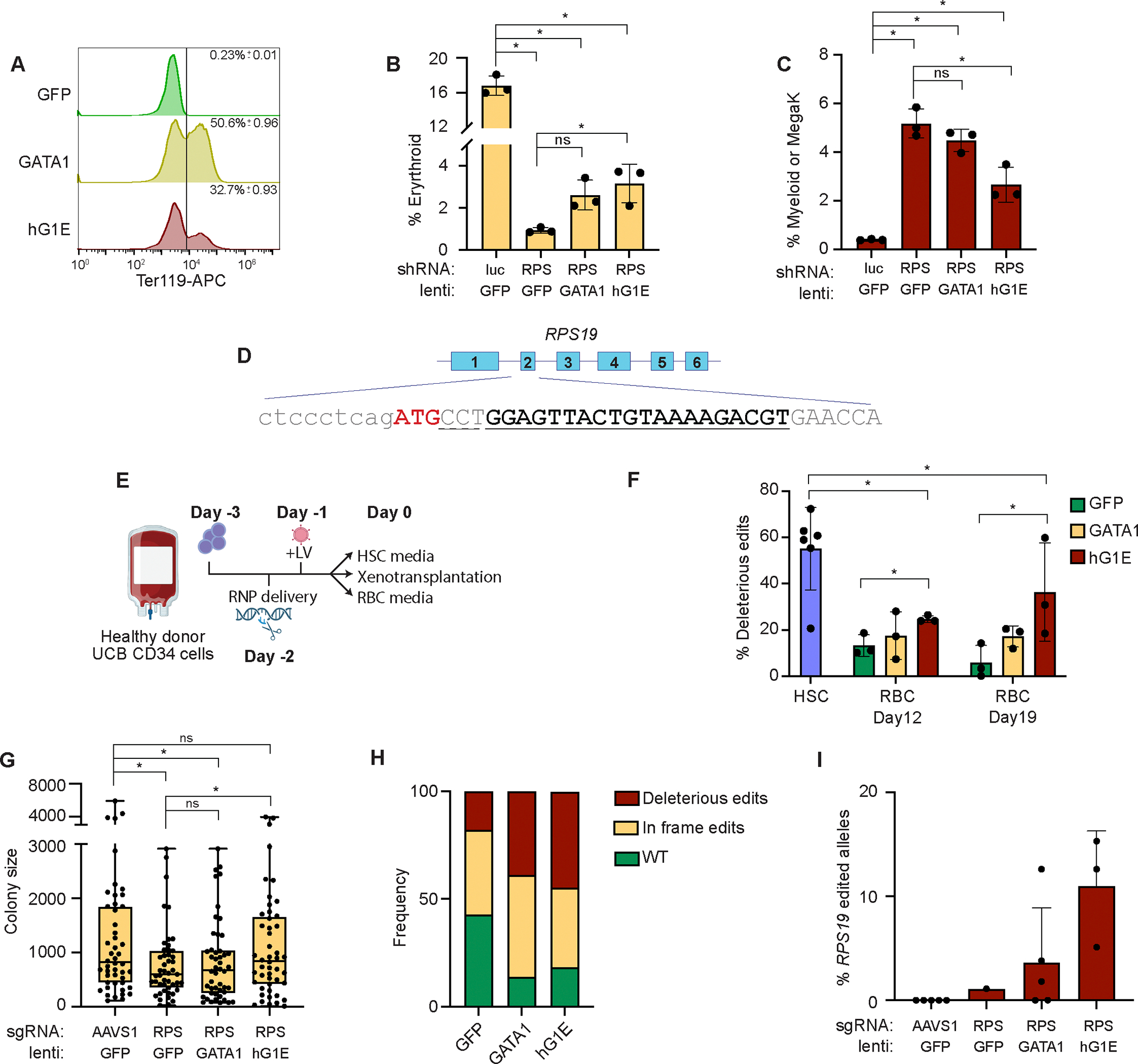
hG1E-GATA1 stimulates erythroid output in DBA models **A.** Differentiation of Gata1^*−/−*^ G1E cells. G1E cells were treated with the indicated vectors and Ter119 expression of GFP^+^ cells was determined by flow cytometry on day 3. Plots include data from three independent replicates, Mean and S.E.M. are displayed. **B.** Rescue of erythroid differentiation after *RPS19* knockdown. Human HSPCs were co-infected with the indicated shRNA lentiviruses targeting luciferase (Luc) or *RPS19* (RPS), and HMD-GFP (GFP), HMD-GATA1 (GATA1), or hG1E-GATA1 (hG1E). Percentage of CD235a^+^ erythroid cells on day 6 of erythroid culture are displayed. n = 3 independent replicates, mean and S.E.M. are shown. Two-sided Student *t*-test was used for comparisons. * P < 0.05, ns - not significant. **C.** Restoration of lineage-skewing after *RPS19* knockdown. Cells from Fig. 3B were stained for myeloid (CD14) or megakaryocyte (CD41a) markers on day 6 of erythroid culture. n = 3 independent replicates, mean and S.E.M. are shown. Two-sided Student *t*-test was used for comparisons. * P < 0.05, ns - not significant. **D.** CRISPR editing of *RPS19*. Exon structure and partial DNA sequence of RPS19 is displayed. sgRNA binding site is indicated in bold and underlined, and the dashed line indicated the PAM site. The ATG start codon is shown in red. Deleterious CRISPR edits are defined as indels causing frameshift or disruption of the ATG. **E.** Schematic of experimental overview. UCB: umbilical cord blood, RNP: CRISPR/Cas9 ribonuclear protein, LV: lentivirus. **F.** Preservation of deleterious *RPS19* edits in bulk culture. On day 6 in HSC culture or the indicated days in erythroid culture, *RPS19* genotyping was performed by PCR and Sanger sequencing. Deleterious edits are defined as in Fig. 3D. GFP: HMD-GFP, GATA1: HMD-GATA1, hG1E: hG1E-GATA1-IRES-GFP. n = 3–6 independent replicates as represented by number of symbols. Mean and S.E.M. are shown. Two-sided Student *t*-test was used for comparisons. * P < 0.05 **G.** Erythroid colony size after CRISPR treatment and lentiviral infection. 500 cells per replicate from the samples in Fig. 3F were plated in methylcellulose and expanded for 12 days. Colonies were imaged and identified using StemVision with manual verification. BFU-E colony size was quantified by determination of pixel density using ImageJ. Each symbol represents an individual burst forming unit – erythroid (BFU-E) colony. RPS: RPS19, GFP: HMD-GFP, GATA1: HMD-GATA1, hG1E: hG1E-GATA1-IRES-GFP. Mean and S.E.M. are shown. Two-sided Student *t*-test was used for comparisons. * P < 0.05, ns - not significant. **H.** Genotyping of erythroid colonies. Genomic DNA was collected from individual BFU-E colonies from the *RPS19* edited samples from Fig. 3G. *RPS19* genotyping was performed by PCR amplification and Sanger sequencing. Frequency of the indicated editing outcomes is displayed for each experimental group treated with the indicated lentivirus. Number of genotyped colonies: GFP: 28, GATA1: 36, hG1E: 38. **I.** Genotyping of human cells after xenotransplantation. Human CD45^+^ cells were purified by FACS from the bone marrows of recipient mice 16 weeks after xenotransplantation with CRISPR-edited and vector-treated human HSPCs. Genotyping of *RPS19* was performed by PCR and Sanger sequencing in samples from mice with human chimerism >1%. Individual mice are represented by the symbols in each group. Mean and S.E.M. are shown. See also Figure S3.

Next, we sought to evaluate the efficacy of hG1E-GATA1 treatment in preclinical models of DBA. Treatment of *Gata1*^*−/−*^ G1E cells^34,35^ with HMD-GATA1 or hG1E-GATA1-IRES-GFP induced erythroid differentiation (Fig. 3A). Knockdown of *RPS19* by shRNA^9,14^ in human HSPCs led to impairment of erythroid differentiation, and lineage skewing that was partially rescued by regulated GATA1 expression (Fig. 3B, C). The incompleteness of the rescue was likely due to the challenges in achieving precise 50% knockdown using this shRNA approach (Fig. S3A).

To more faithfully model DBA in primary human HSPCs, we used CRISPR/Cas9 editing to approximate *RPS19* haploinsufficiency (Fig. 3D, E, Fig. S3B). Bulk genotyping of cells kept in HSC maintenance media for 6 days revealed 55% deleterious *RPS19* edits (Fig. 3F). Edited cells transduced with hG1E-GATA1-IRES-GFP (Fig. S3C), in contrast to HMD-GFP, had preservation of deleterious *RPS19* edits, revealing that regulated GATA1 expression can overcome *RPS19* deficiency and support erythroid differentiation (Fig. 3F).

In colony forming unit (CFU) assays^25^ (Fig. S3D), *RPS19* editing led to smaller mean colony size (indicating fewer progeny from each erythroid progenitor^36^) which was rescued by treatment with hG1E-GATA1-IRES-GFP (Fig. 3G, Fig. S3E). We did not observe the same rescue in the HMD-GATA1 treated samples, perhaps due to the detrimental effects of constitutive GATA1 expression during terminal erythroid differentiation. Unlike HMD-GFP samples, hG1E-GATA1-IRES-GFP treated colonies had preservation of deleterious *RPS19* edited alleles (Fig. 3H), showing at the individual progenitor level that regulated GATA1 expression can overcome RPS19 deficiency.

Next, we performed xenotransplantation into NBSGW mice of human HSPCs after *RPS19* disruption and hG1E-GATA1-IRES-GFP treatment. Compared to controls, *RPS19* edited cells had significantly lower human chimerism at 16 weeks regardless of further treatment, which limited our ability to perform rigorous comparisons of erythroid progenitors (Fig. S3F, G). We observed partial preservation of deleterious *RPS19* edits only in the samples treated with hG1E-GATA (Fig. 3I).

We hypothesized that the incomplete rescue in our CRISPR models was due to detrimental impacts of biallelically edited cells. We observed a reduction in myeloid colonies (in which heterozygous loss of *RPS19* is expected to be tolerated^10^) (Fig. S3H), suggesting that biallelic deleterious edits were present but selected against. Genotyping of myeloid colonies revealed that 50% of myeloid progenitors had biallelic editing with at least one in-frame edit (Fig. S3I). Although some of these biallelically edited progenitors were able to generate colonies *in vitro*, the profound engraftment disadvantage of *RPS19* edited cells that we and others^10^ observed is consistent with a broad hematopoietic defect following biallelic *RPS19* perturbation that is related to the heterogenous nature of CRISPR editing. Together, these data show that regulated GATA1 expression is sufficient to overcome the erythroid maturation defect caused by *RPS19* haploinsufficiency.

### Regulated GATA1 expression improves erythroid output in DBA patient samples *in vitro*

**Figure 4.**
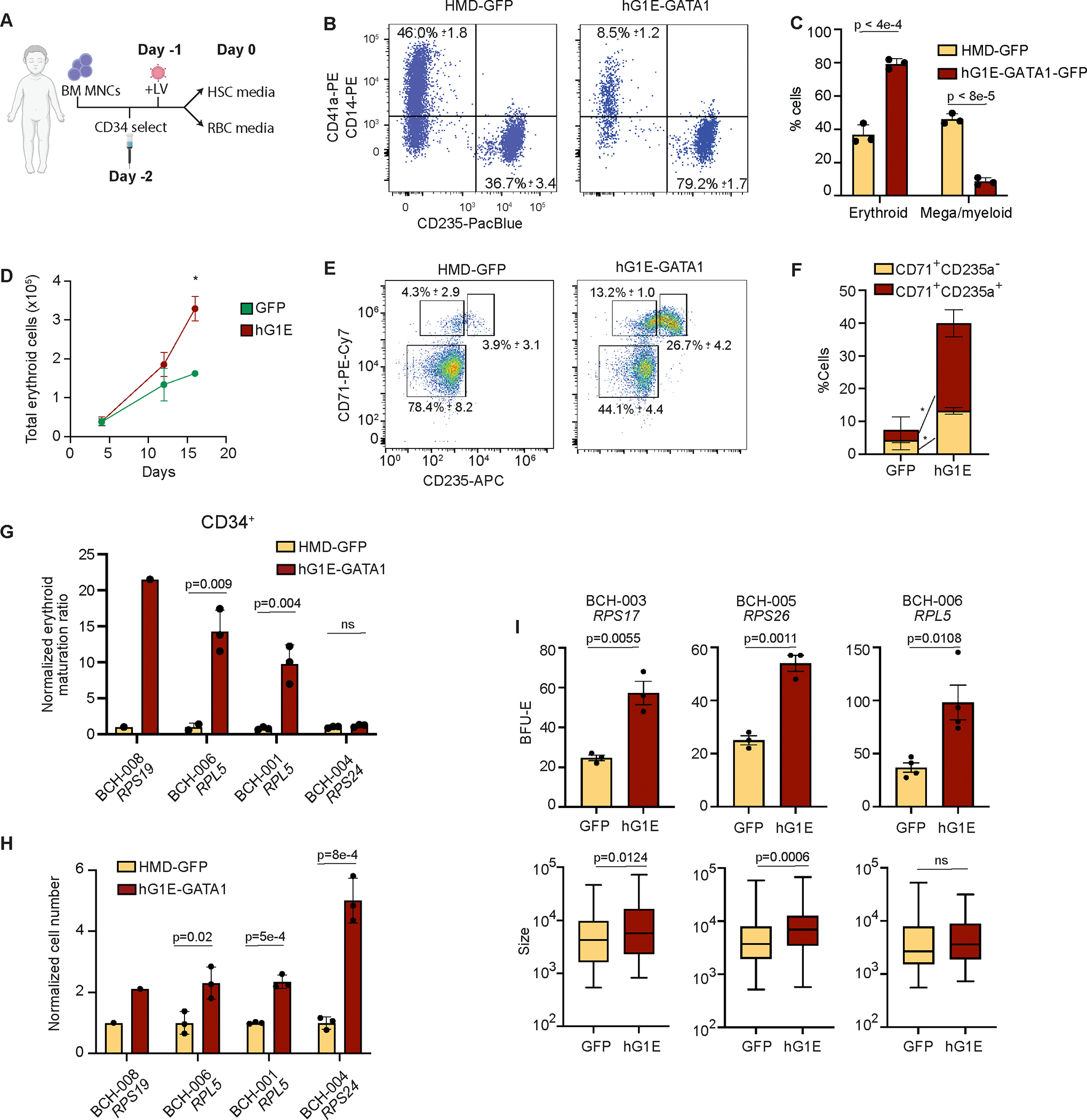
Stimulation of erythroid output in primary DBA patient samples **A.** Schematic of experimental overview. BM MNC: bone marrow mononuclear cells, LV: lentivirus. **B.– C.** *In vitro* erythroid differentiation of DBA patient sample. CD34-selected HSPCs from patient BCH-001 were treated with the indicated vector and cultured in erythroid differentiation media. On day 16 of erythroid culture, samples were analyzed for expression of erythroid marker CD235a or megakaryocyte (CD41a) and myeloid (CD14) markers. Mean and S.E.M. are shown. Two-sided Student *t*-test was used for comparisons. P values are shown. **D.** Total erythroid cell number. Erythroid cell number (calculated by multiplying total cell number by percent cells expressing CD71, CD235a or both) was quantified using Trypan blue exclusion on the indicated days. Mean and S.E.M. are shown. Two-sided Student *t*-test was used for comparisons. * P = 0.008. Absent error bars are obscured by the size of the markers. **E.- F.** Erythroid differentiation of gene therapy treated sample from DBA patient BCH-006. On day 7 of erythroid culture, stage of erythroid differentiation was assessed by analysis of surface expression of CD71 and CD235a. Mean and S.E.M. from three biological replicates are displayed. Two-sided Student *t*-test was used for comparisons. * P < 0.008. **G.** Normalized erythroid ratio following hG1E-GATA1 treatment. On day 6 of erythroid culture, the erythroid maturation ratio was calculated by dividing the percentage of CD71^+^CD235a^+^ cells by the percentage of CD71^+^CD235a^−^ cells and was then normalized to the erythroid maturation ratio of the HMD-GFP treated control. Number of markers represents the number of replicates (1 or 3). Mean and S.E.M. are shown where appropriate. Two-sided Student *t*-test was used for comparisons. P values are shown, ns – not significant. **H.** Total cell number during erythroid differentiation. Total cell number was quantified from CD34-selected DBA patient samples treated with the indicated vectors using Trypan blue exclusion on day 16 and normalized to HMD-GFP treated sample from the same patient. Mean and S.E.M. are shown where appropriate. Two-sided Student *t*-test was used for comparisons. P values are shown. **I.** Quantification of erythroid colony number and size. BM MNCs from the indicated patient samples were treated with HMD-GFP or hG1E-GATA1 and 30,000 cells per replicate were plated in methylcellulose. On day 12 of methylcellulose culture, burst forming-erythroid (BFU-E) colonies were quantified by StemVision. Colony size was measured by pixel density using ImageJ. Colony number (top) was determined as the mean of 3 or 4 independent replicates as shown and is displayed with S.E.M. Mean colony size (bottom) is displayed with S.E.M. Two-sided Student *t*-test was used for comparisons. P values are shown, ns – not significant. See also Figure S4 and Table S1.

We next tested our gene therapy vector in samples from primary DBA patients. First, we treated CD34^+^ HSPCs from patient BCH-001, who has an *RPL5* mutation, with either HMD-GFP or hG1E-GATA1-IRES-GFP (Fig. 4A). We observed erythroid-restricted transgene expression without impairment in phenotypic LT-HSCs (Fig. S4A, B). Almost two-thirds of cells in the HMD-GFP group were unable to bypass the erythroid maturation arrest and express CD235a (Fig. 4B), instead expressing myeloid or megakaryocyte markers (Fig. 4C), highlighting the disordered hematopoiesis seen in DBA patient progenitors. In contrast, almost 80% in the hG1E-GATA1-IRES-GFP group underwent erythroid differentiation (Fig. 4B–D, Fig. S4C, D).

Next, we obtained CD34^+^ HSPCs from three additional DBA patients of varied genotypes (Table S1) and treated them with either hG1E-GATA1 or HMD-GFP. hG1E-GATA1 treatment of CD34^+^ HSPCs from BCH-006 resulted in stimulation of erythroid differentiation with a higher proportion of cells able to overcome erythroid maturation arrest (Fig. 4E, F, Fig. S4E), leading to an increase in total cell number (Fig. S4F). In three out of four DBA patient samples, the erythroid maturation ratio (CD235a^+^ cells divided by total CD71^+^ cells) was significantly higher after hG1E-GATA1 treatment (Fig. 4G). In patient BCH-004, there was no increase in the erythroid maturation ratio, but there was a 5-fold increase in total erythroid cell number, showing that hG1E-GATA1 treatment in this patient sample supported increased expansion throughout erythroid maturation (Fig. 4H). These results reveal that hG1E-GATA1 treatment of primary HSPCs from DBA patients is sufficient to overcome the erythroid maturation block and increase erythroid output.

Next, we obtained primary bone marrow mononuclear cells (MNCs) from a total of 12 DBA patients of varied genotypes (Table S1), which account for ~86% of all genetically resolved cases of DBA^7^ (Fig. S4G). Because of limited cell numbers, we could not perform CD34-selection but rather treated whole bone marrow MNCs (which included HSPCs, CD71^+^ erythroid progenitors, and other lineage precursors) followed by erythroid differentiation. Despite the lineage heterogeneity, hG1E-GATA1 stimulated increased erythroid maturation irrespective of genotype (Fig. S4H). We directly compared the magnitude of effect sizes in the CD34-selected HSPCs with unselected MNCs in the three patients with sufficient cell number that enabled comparable analyses (Fig. S4H, red text), and found a much more modest increase in the erythroid maturation ratio in the unselected MNC samples. These results indicate that the increase in erythroid maturation ratio of DBA patient MNCs underestimates the true impact of hG1E on erythroid differentiation. Additionally, patients with mutations in the same gene had variable increases in erythroid maturation following hG1E-GATA1 treatment, which might be due to variable mutation effects on phenotype or differences in erythroid progenitor number across patients.

To further characterize the improved erythroid output in primary DBA samples, we performed CFU assays after hG1E-GATA1 or HMD-GFP treatment. The DBA patient samples showed no impairment in maturation of myeloid progenitors (Fig. S4I), but demonstrated increases in the number and size of erythroid colonies after hG1E-GATA1 treatment (Fig. 4I). The limited sample quantity from patient BCH-003 (*RPS17* mutation) had only a slight increase in erythroid maturation ratio in bulk culture (Fig. S4H), but showed significantly more and larger colonies in the CFU assay (Fig. 4I), further emphasizing that the erythroid maturation ratio in bone marrow MNCs underestimates the true impact on erythroid differentiation. Collectively, these data reveal that hG1E-GATA1 gene therapy treatment of DBA patient samples stimulates increased erythroid differentiation *in vitro* regardless of genotype.

### Gene therapy with hG1E-GATA1 stimulates erythroid differentiation in DBA patient samples *in vivo*

**Figure 5.**
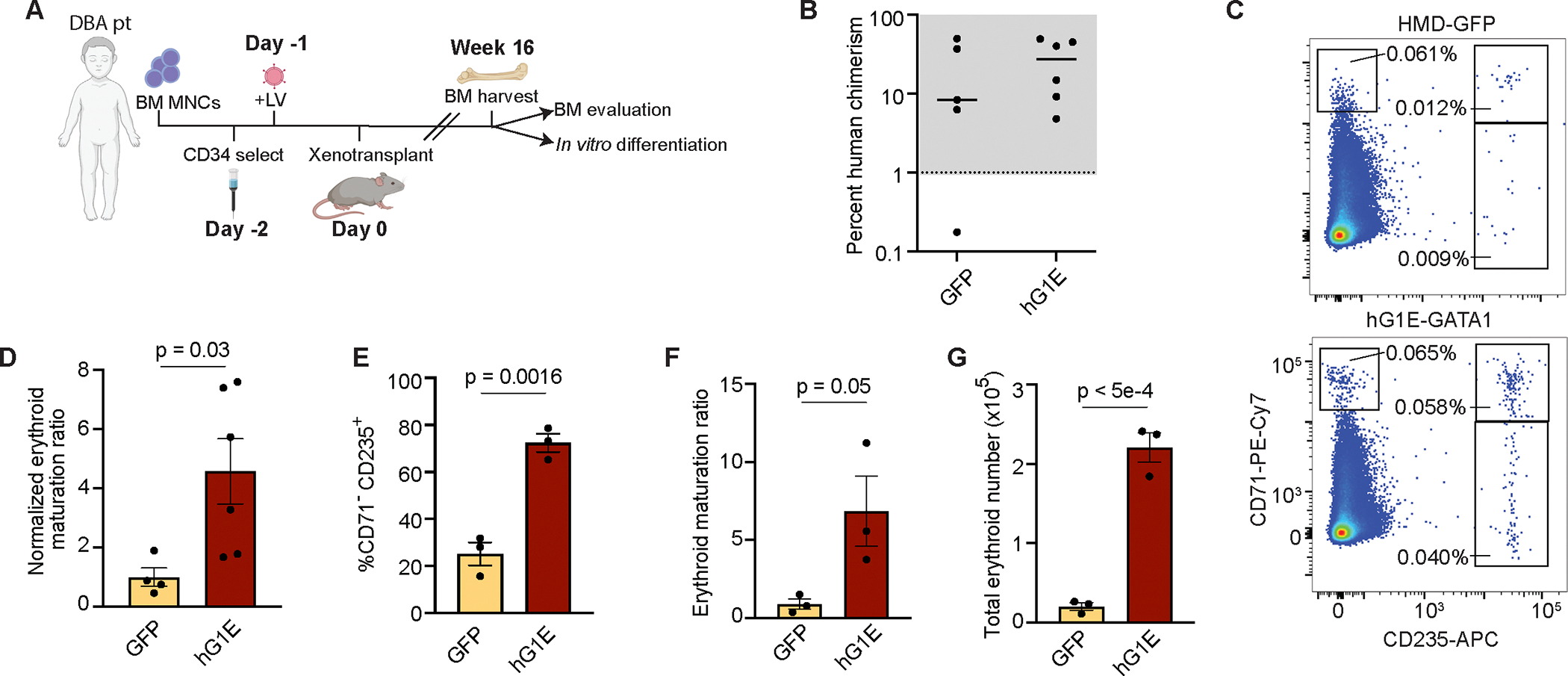
Increased erythroid output in primary DBA patient samples *in vivo* **A.** Schematic of experimental overview. BM MNC: bone marrow mononuclear cells, LV: lentivirus. **B.** Evaluation of human chimerism. After xenotransplantation with CD34-selected and gene therapy treated cells from DBA patient BCH-006, human chimerism in the bone marrow was determined by comparing the percentage of human CD45^+^ cells to the percentage of mouse CD45^+^ cells by flow cytometry. Each marker represents one recipient mouse. Mice with human chimerism >1% were used in subsequent analyses. **C.** Representative flow cytometry plots of *in vivo* human erythroid differentiation in the bone marrow of recipient mice. Whole bone marrow cells harvested at 16 weeks without erythrocyte depletion were stained with the indicated human-specific antibodies and analyzed by flow cytometry. Committed erythroid progenitors have high expression of CD71 and maturing erythroid progenitors express CD235. **D.** Increased erythroid maturation *in vivo*. Erythroid maturation ratio was calculated by dividing the percentage of CD235a^+^ cells by the percentage of CD71^+^CD235a^−^ cells and was then normalized to the erythroid maturation ratio of the HMD-GFP treated control. Number of markers represents the number of replicates. Mean, S.E.M., and P value are shown. Two-sided Student *t*-test was used for comparisons. **E.- G.** Erythroid output of xenotransplanted samples. Human CD34^+^ HSPCs from the indicated primary xenotransplant cohorts were combined and subjected to *in vitro* erythroid differentiation. Percentage of CD71^−^CD235a^+^ cells on day 21 are shown (**E.**). Erythroid maturation ratio was calculated by dividing the percentage of CD235a^+^ cells by the percentage of CD71^+^CD235a^−^ cells and was then normalized to the erythroid maturation ratio of the HMD-GFP treated control (**F.**). Total erythroid number was calculated by multiplying total cell number (Fig. S5D) by the percentage of cells expressing CD235a on day 21 (**G.**). n = 3 independent replicates in *in vitro* culture. Mean, S.E.M., and P values are shown. Two-sided Student *t*-test was used for comparisons. See also Figure S5.

To assess the impact of hG1E-GATA1 treatment of DBA patient samples *in vivo*, CD34^+^ HSPCs from *RPL5* patient BCH-006 were treated with HMD-GFP or hG1E-GATA1, followed by xenotransplantation into NBSGW mice (Fig. 5A). hG1E-GATA1 treatment had no detrimental effect on human cell engraftment (Fig. 5B) or the proportion of non-erythroid cells (Fig. S5A). We found no difference in the percentage of erythroid committed CD71^+^ progenitors (Fig. S5B), but there was an increase in erythroid maturation ratio in the samples treated with hG1E-GATA1 (Fig. 5C, D). To corroborate these *in vivo* data, we performed xenotransplantation of hG1E-GATA1 or control treated whole bone marrow MNCs from *RPL5* patient BCH-001 and observed a significant increase in erythroid maturation ratio in the hG1E-GATA1 group (Fig. S5C).

We next purified human CD34^+^ HSPCs from the bone marrows of recipient mice that had been transplanted with treated CD34^+^ HSPCs from patient BCH-006. We reasoned that if hG1E-GATA1 treatment had a detrimental impact on HSC engraftment, this population of CD34^+^ HSPCs would be enriched for non-transduced cells that would not be able to increase erythroid output *in vitro* beyond that of control treated samples. Instead, we found that CD34^+^ progenitors harvested from hG1E-GATA1 recipient bone marrows generated significantly higher percentages of mature CD71^−^CD235a^+^ erythrocytes *in vitro* (Fig. 5E). The erythroid maturation ratio was 8.5-fold higher (Fig. 5F). There was also an increase in the total cell number (Fig. S5D), and the total number of mature erythrocytes was more than 11-fold higher (Fig. 5G). To corroborate these findings, we purified human CD71^+^ erythroid progenitors from xenotransplant recipients, and observed increased erythroid output (Fig. S5E, F) in *in vitro* culture. These data reveal that hG1E-GATA1 treatment improves erythroid maturation in DBA patient samples *in vivo* and does not cause detectable impairment of transduced HSCs.

### Regulated GATA1 expression reverses transcriptional signatures of erythropoietic stress in DBA

**Figure 6.**
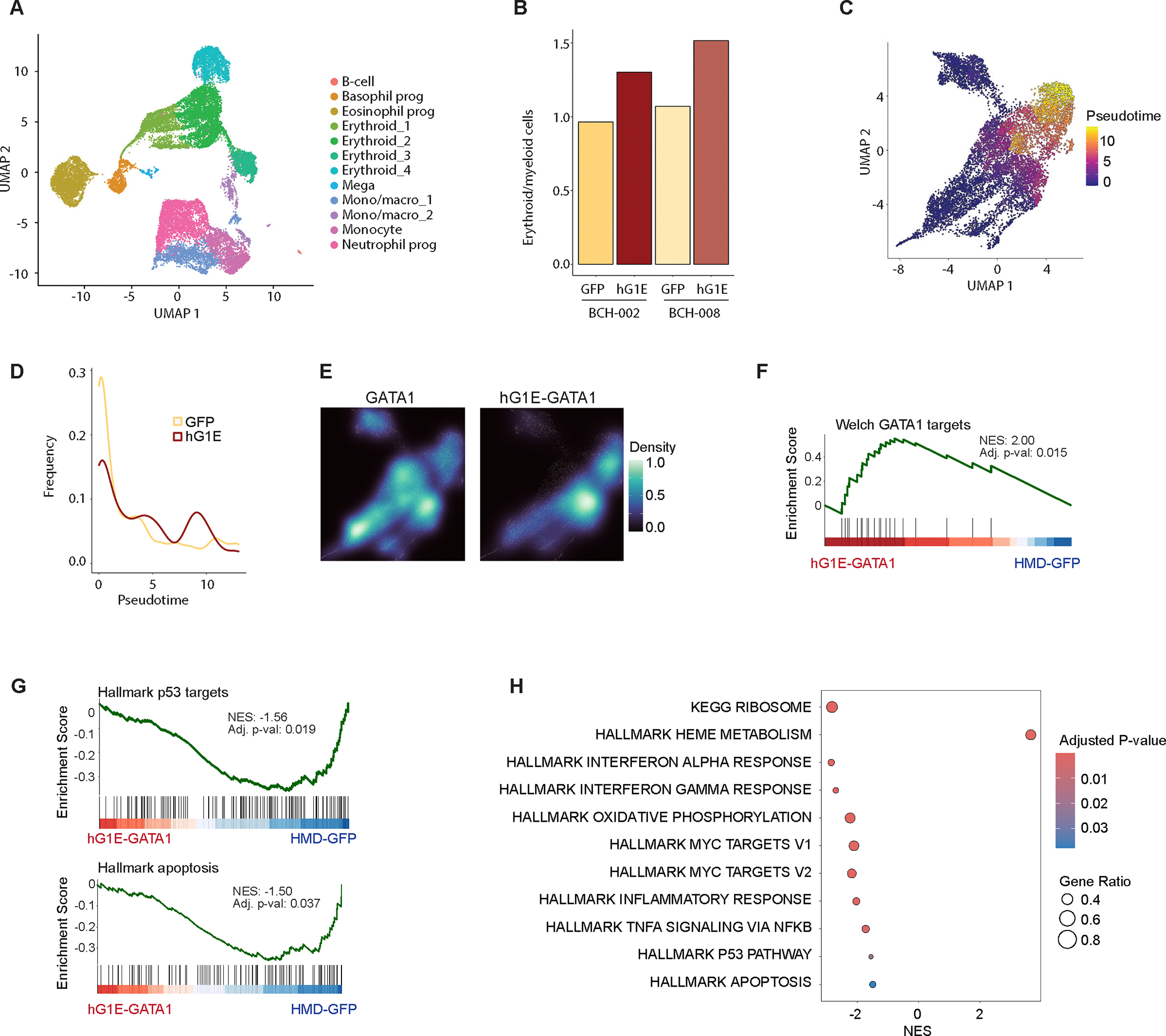
Reversal of transcriptional dysregulation upon hG1E-GATA1 treatment **A.** Uniform manifold approximation and projection (UMAP) projection of cultured cells from DBA patients BCH-002 and BCH-008 after treatment with HMD-GFP or hG1E-GATA1, at day 10 of *in vitro* erythroid differentiation. Clusters were annotated based on the expression of the top 10 marker genes. **B.** Ratio of erythroid cells to myeloid cells as determined by transcriptional signatures of single cells from DBA patients after HMD-GFP or hG1E-GATA1 treatment. **C.** UMAP plot of erythroid-filtered cells colored by pseudotime trajectory indicating the degree of erythroid maturation. **D.** Density of erythroid cells ordered along the pseudotime axis following HMD-GFP or hG1E-GATA1 treatment. **E.** UMAP projection of the density estimate of erythroid cells expressing endogenous GATA1 (left) or hG1E-GATA1 transgene (right). The displayed density plots are derived from the same erythroid-filtered UMAP projections shown in Fig. 6C and Fig. S6B, C. **F.- G.** Gene set enrichment analysis (GSEA) plots showing (**F.**) enrichment of GATA1 target genes and (**G.**) depletion of Hallmark p53 pathway genes (top) and Hallmark apoptosis genes (bottom) in erythroid cells following hG1E-GATA1 treatment. Normalized enrichment score (NES) and p-value are shown. The Kolmogorov Smirnov (K-S) test was used to determine the significance of GSEA. **H.** Bubble plot of selected pathways from the Hallmark and KEGG collections that are differentially expressed in hG1E-GATA1 treated DBA patient erythroid cells. Normalized enrichment score (NES) is displayed on the x-axis. The color represents the adjusted P value, and the size of the bubbles shows the Gene ratio, defined as the proportion of differentially expressed genes relative to the size of the gene set. See also Figure S6.

Next, we evaluated the transcriptional impact of hG1E-GATA1 treatment on DBA patient hematopoietic progenitors undergoing erythroid differentiation. We transduced CD34^+^ HSPCs from DBA patients BCH-002 (with a mutation in *RPL35A*) and BCH-008 (with a mutation in *RPS19*) with HMD-GFP or hG1E-GATA1 and maintained the cells in erythroid differentiation media for 10 days prior to collection for single-cell RNA sequencing (scRNA-seq). Consistent with our earlier flow cytometry data, scRNA-seq revealed the presence of multiple hematopoietic lineages (Fig. 6A, Fig. S6A). hG1E-GATA1 treatment resulted in higher erythroid cell proportions in both DBA patients (Fig. 6B). Pseudotime trajectories of erythroid committed cells (Fig. 6C, Fig. S6B, C) revealed that hG1E-GATA1 expressing cells were significantly further along the differentiation trajectory, consistent with amelioration of the impaired differentiation observed in DBA (Fig. 6D, Fig. S6D, E).

We compared endogenous GATA1 expression to codon-optimized hG1E-GATA1 expression (Fig. S6F) in erythroid cells. While endogenous GATA1 expression occurred throughout erythroid differentiation, hG1E-GATA1-expressing cells were enriched at the later stages of differentiation (Fig. 6E). Since the transcriptional regulation of endogenous GATA1 and hG1E-GATA1 expression is similar, this result suggests that those progenitors with exogenous GATA1 expression are better able to undergo erythroid differentiation, not that there is simply altered expression.

Next, we examined gene expression profiles in erythroid progenitors expressing hG1E-GATA1, and found significant upregulation of GATA1 target genes (Fig. 6F). Compared to healthy donor controls, erythroid progenitors in DBA have upregulation of gene sets related to p53, apoptosis, inflammation, ribosomal proteins, and MYC signaling^36–38^. hG1E-GATA1 expression in DBA patient cells led to a significant downregulation of these gene sets (Fig. 6G, H, Fig. S6G), consistent with reversal of the transcriptional signatures of ineffective erythropoiesis. Accumulation of excess heme leads to downregulation of heme synthesis in DBA^36,37^, and we observed upregulation heme metabolism genes in hG1E-GATA1 expressing cells (Fig. 6H). Together, we demonstrate that hG1E-GATA1 treatment of primary DBA patient samples reverses the erythroid transcriptional dysregulation that is characteristic of DBA.

### Safe genomic integration of hG1E-GATA1

**Figure 7.**
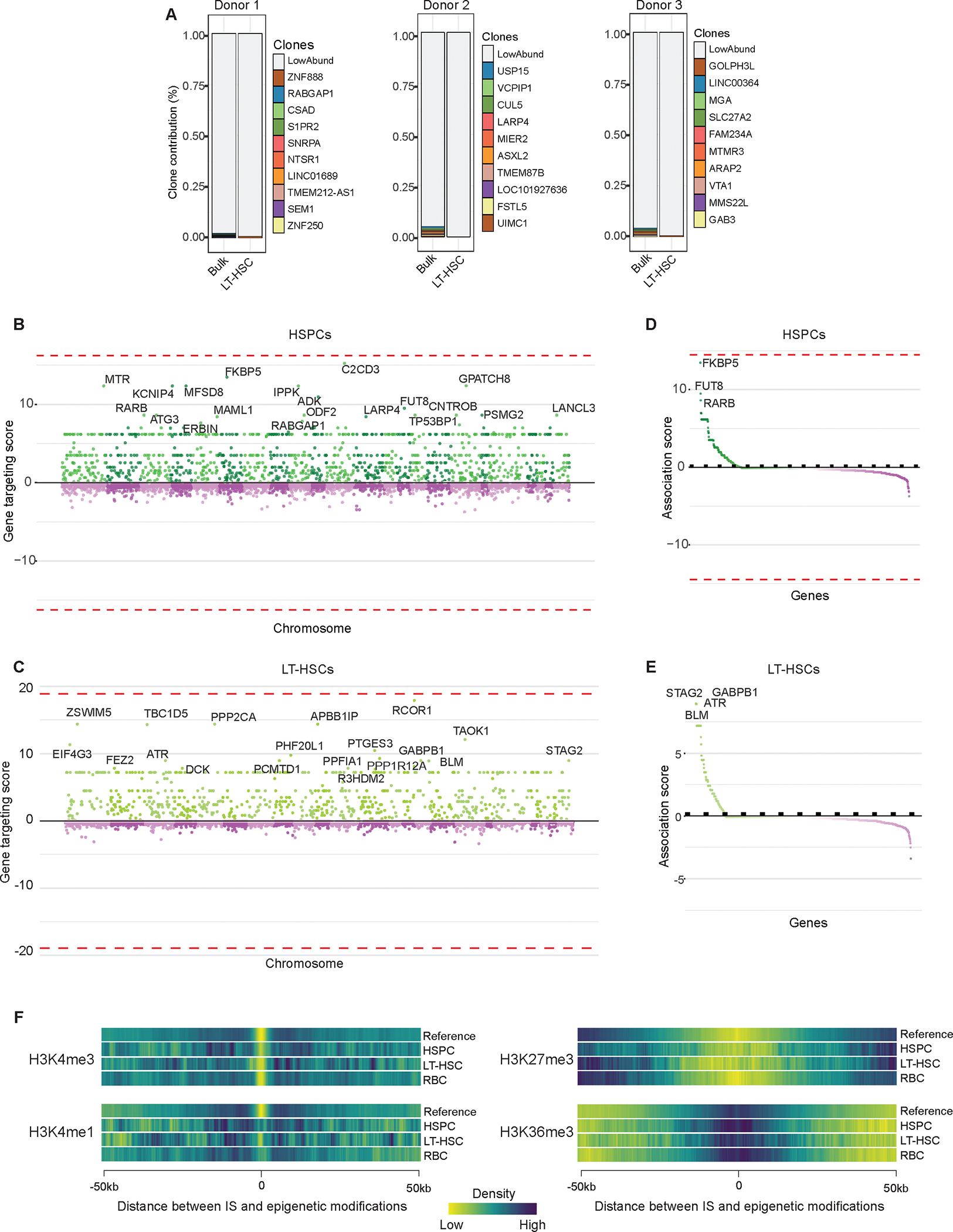
Analysis of integration sites of hG1E-GATA1 **A.** Cumulative frequencies of integration sites of hG1E-GATA1 following transduction in bulk HSPCs and in LT-HSCs on day 7 of *in vitro* culture from three separate healthy donors. RefSeq names of the genes closest to integration sites are displayed. **B.- C.** Comparison of integration sites of hG1E-GATA1 in bulk HSPCs (**B.**) and LT-HSCs (**C.**). Integration sites were compared to the integration profile of a control lentivirus^40^ in HSPCs and sites are organized by chromosome location along the x-axis. Relative gene targeting score is shown on the y-axis. No integration sites are significantly enriched in hG1E-GATA1 treated samples (dotted lines). **D.- E.** Integration near cancer associated genes. Gene targeting scores of cancer-associated genes^41^ near hG1E-GATA1 integration events in bulk HSPCs (**D**.) and LT-HSCs (**E**.) are shown. No integration sites are significantly enriched in the hG1E-GATA1 treated samples (dotted lines). **F.** Heatmaps of the epigenetic landscape of hG1E-GATA1 integration sites. Integration sites from hG1E-GATA1 and a reference lentivirus^40^ are displayed as a function of chromosomal distance from epigenetic modifications derived from ChIP-seq data in human CD34^+^ cells^42^ Epigenetic modifications represent the following chromosomal features: H3K4me3 – active promoter, H3K4me1 – active enhancers, H3K27me3 – repressed enhancers and promoters, H3K36me3 – actively transcribed gene bodies. See also Figure S7 and Table S2.

Finally, to generate a full pre-clinical dataset to enable human clinical studies, we performed integration site analysis (ISA)^39^ of hG1E-GATA1 in human hematopoietic cells. We found a diverse clonal repertoire of integration sites following transduction of CD34^+^ cells from three separate healthy human donors with hG1E-GATA1 (Fig. 7A, Table S2), both in the bulk HSPCs and in the purified population of LT-HSCs after *in vitro* culture. Compared to the integration profile of an alternative lentiviral gene therapy vector^40^, we did not observe any integration events at significantly increased frequency (Fig. 7B, C). In particular, there was no predilection for integration near known cancer associated genes^41^ (Fig. 7D, E). The epigenetic landscape^42^ of integration sites was similar to other lentiviral gene therapy vectors^40^ with enriched frequency near actively transcribed regions (Fig. 7F).

To examine clonal dynamics during *in vitro* erythroid differentiation, we performed ISA on a post-transduction sample in HSC culture and again on day 18 of erythroid culture (Fig. S7A). There was not significant enrichment for any integration events in the erythroid samples compared to the integration profile of an alternative lentiviral product^40^ (Fig. S7B).

Finally, to examine clonal dynamics *in vivo*, we performed ISA in human CD45-selected cells harvested from the bone marrow of mice transplanted with hG1E-GATA1 treated cells from DBA patient BCH-006 (Fig. 5A–5E) or healthy donor (Fig. 2G). From limited sample material, we detected integration events in 2,749 cells and found no integration events at genomic loci associated with clonal expansion after gene therapy near *LMO2*, *IKZF1*, *CCND2*, *HMGA2,* or *MECOM,* and no overrepresentation near cancer-related genes. Together, these data reveal that our clinical-grade hG1E-GATA1 lentiviral vector has a genomic integration profile that approximates what has been observed in other lentiviral gene therapy products^40^, and provide strong support for the clinical translation of regulated GATA1 as a universal gene therapy for DBA.

## Discussion

Since the discovery of *RPS19* mutations as the first genetic cause of DBA^43^, gene therapy has been an attractive platform for curative therapy^44–46^. However, efforts have focused on *RPS19* gene addition, that if successfully translated into clinical use, would only be applicable to the minority of patients that have RPS19 haploinsufficiency, leaving most DBA patients with insufficient treatment options. Developing and testing gene replacement vectors for each of the more than thirty genes implicated in DBA is not feasible. Instead, we have designed a universal gene therapy vector applicable to all patients with DBA that harnesses endogenous regulatory sequences to achieve erythroid-specific expression of GATA1. We have demonstrated that hG1E-GATA1 gene therapy increases erythroid output and reverses pathologic transcriptomic changes in primary DBA patient samples, without compromising HSC function.

These pre-clinical efficacy and safety data provide support to initiate what will be the first-in-human gene therapy trial of regulated GATA1 expression as a universal treatment for Diamond-Blackfan anemia. More broadly, our study provides a key proof-of-concept for the functional correction of a genetic hematopoietic disorder by augmenting expression of a downstream target rather than repairing or replacing the mutated gene itself, establishing a new paradigm for gene therapies targeting HSCs. By focusing on conserved downstream mechanisms rather than specific, but individually rare, disparate mutations, this approach will broaden the reach of hematopoietic gene therapies. We envision that similar strategies can be developed to treat other rare or genetically complex conditions that are not currently conducive to traditional gene therapy approaches.

## Limitations of this study

While our data are promising, they cannot directly address the extent to which the stimulation of erythropoiesis we observe would impact the anemia in DBA patients following gene therapy. However, useful inferences can be made from published data. Our observed effect size of up to 21-fold stimulation of erythroid production is better than reported effect sizes in the setting of *RPS19* gene addition. Hamaguchi and colleagues reported a 2–3 fold increase in BFU-E colonies in DBA patient samples treated with an RPS19-IRES-GFP lentiviral cassette and sorted for high GFP positivity^45^. Bhoopalan and colleagues demonstrated a 2-fold increase in BFU-E number in a CRISPR-based human HSPC model of *RPS19* haploinsufficiency following lentiviral *RPS19* overexpression, and showed an approximately 2-fold increase in CD235a^+^ erythroid cells *in vivo*^10^. Giménez and colleagues showed a 2–4-fold increase in terminally differentiated CD71^−^ CD235^+^ erythroid cells from RPS19 haploinsufficient DBA patients following lentiviral RPS19 gene addition^47^. Perhaps even more relevant is the quantification of erythroid output in *in vitro* generated DBA models following corticosteroid treatment, as this modality is at least partially effective in most DBA patients. Narla and colleagues demonstrated a 3–4-fold increase in total erythroid production *in vitro* following dexamethasone treatment of shRPS19 treated primary HSPCs^48^. It remains to be seen whether the 11-fold increase in total erythroid cells we observed following hG1E-GATA1 treatment (as in Fig. 5G) will translate to a similar (or greater) magnitude of effect *in vivo* in patients after gene therapy treatment. This can only be conclusively answered through a clinical trial.

## Resource Availability

### Lead contact

Further information and requests for resources and reagents should be directed to and will be fulfilled by the lead contact, Vijay G. Sankaran (vijay.sankaran@childrens.harvard.edu)

### Materials availability

All unique/stable reagents generated in this study are available from the lead contact without restriction.

### Data and code availability

The single-cell RNA sequencing data are deposited in National Center for Biotechnology Information Gene Expression Omnibus with the identifier GSE261450 and are publicly available as of the date of publication. This paper does not report original code. Any additional information required to reanalyze the data reported in this paper is available from the lead contact upon request.

## STAR Methods

### EXPERIMENTAL MODELS

#### Cell line and primary cell culture

Healthy donor HSPCs were purified from discarded umbilical cord blood samples of healthy male or female newborns collected by the Pasquarello Tissue Bank at Dana-Farber Cancer Institute (IBC-P00000180) using the EasySep Human CD34 Positive Selection Kit II following pre-enrichment using the RosetteSep Pre-enrichment cocktail (Stem Cell Technologies, 17896) and mononuclear cell isolation on Ficoll-Paque (GE Healthcare, 17-1440-02) density gradient. Cells were cryopreserved for later use. G-CSF mobilized adult CD34^+^ HSPCs from de-identified males and females were purchased (Fred Hutchinson Cancer Research Center) and stored in liquid nitrogen until use. DBA patient bone marrow samples were collected after informed consent was granted following research protocol guidelines approved by IRB at Boston Children’s Hospital, Cincinnati Children’s Hospital and Children’s Hospital Colorado and MNCs were cryopreserved. Thawed cells were cultured at 37°C in serum-free HSC media comprised of StemSpan II medium (Stem Cell Technologies, 09605) supplemented with CC100 cytokine cocktail (Stem Cell Technologies, 02690), 100ng/ml TPO (Peprotech, 300–18) and 35nM UM171 (Stem Cell Technologies, 72912). Confluency was maintained between 2e5–1e6 cells/ml. CD34-selection of DBA patient bone marrow MNCs was performed using EasySep Human CD34 Positive Selection Kit II.

Erythroid differentiation of primary HSPCs was performed in a three-phase culture system as we have previously described^50^. Cells were maintained at a concentration of 1e5–1e6/mL and media was refreshed or replace every 3–4 days with the following media: Phase I (day 0–7): IMDM (Life Technologies, 12440–061) supplemented with 3% human AB serum (Atlanta Biologicals, S40110), 2% human AB plasma (SeraCare, 1810–0001), 1% penicillin/streptomycin (Life Technologies, 15140–122), 10 μg/mL insulin (Lilly, NDC 0002-8215-01), 3 IU/mL heparin (Hospira, NDC 00409-2720-01), 200 μg/mL holo-transferrin (Sigma-Aldrich, T0665), 10 ng/mL stem cell factor (SCF) (Peprotech, 300–07), 1 IU/mL erythropoietin (EPO) (Amgen, NDC 55513-267-10) and 1 ng/mL IL-3 (Peprotech, 200–03). Phase II (days 8–13): IL-3 was omitted from the medium. Phase III (days 14–21): IL-3 and SCF were omitted, and holo-transferrin concentration was adjusted to 1mg/mL.

For colony-forming assays, 500 HSPCs or 30,000 BM MNCs were plated in MethoCult H4434 (StemCell Technologies, 04434), in three 35mm dishes (technical replicates) for sample and grown at 37°C with 5% CO_2_ for 12 days. Colonies were imaged using StemVision (StemCell Technologies) and colony size was determined using density of pixels in each colony using ImageJ. For serial CFU assays, 50,000 unselected MNCs harvested from primary xenotransplant recipients were plated in MethoCult H4434 prior to imaging on day 9 and subsequent replating of 50,000 cells per replicate. This was performed for four rounds of replating at which time there were no colonies formed in either control or experimental groups.

293T cells were cultured at 37°C in DMEM (Life Technologies, 11965–118) supplemented with 10% FBS (BioTechne, S11550), 1% l-glutamine (Thermo, 25-030-081) and 1% penicillin/streptomycin (Life Technologies, 15140–122).

G1E cells were cultured at 37°C in IMDM supplemented with 15% FBS, 1% penicillin/streptomycin, 50ng/mL murine stem cell factor (Peprotech, 250–03), 2 U/mL human erythropoietin, and 45mM 1-thioglycerol (Sigma M6145) as previously described^12^.

#### Mouse model

NOD.Cg-*Kit*^W−41J^*Tyr*^+^*Prkdc*^scid^*Il2rg*^tm1Wjl^(NBSGW) mice were obtained from Jackson Laboratory (RRID: IMSR_JAX:026622)^19^. Mice of the same sex were randomly assigned to experimental or control groups. NBSGW were interbred to maintain a colony of animals homozygous or hemizygous for all mutations of interest. Mice of the same sex were housed 5 to a cage and provided a standard chow diet. Standard light/dark cycles of 12 hours each were maintained. Autoclaved sulfatrim antibiotic water was provided and changed weekly to minimize the risk of infection. The Institutional Animal Care and Use Committee (IACUC) at Boston Children’s Hospital approved the study protocol and provided ethical oversight.

Non-irradiated NBSGW mice (between 4–8 weeks of age) were tail vein injected with UCB, adult CD34^+^ HSPCs (1–2e5 cells) or bone marrow MNCs (0.8–1e6 cells) on day 1 after viral transduction. Animals were sacrificed at 16 weeks for bone marrow evaluation. For secondary xenotransplants, cryopreserved total bone marrow from 10 primary recipient mice in each cohort was thawed and combined and CD34^+^ cells were selected using EasySep Human CD34 Positive Selection Kit II (Stem Cell Technologies). 2e5 cells were transplanted by tail vein injection into 8-week-old NBSGW mice and bone marrow was harvested at 16 weeks for analysis.

### METHOD DETAILS

#### Flow cytometry and cell sorting

Cells were washed with PBS and stained with the following panel of antibodies to quantify and enrich for LT-HSCs: anti-CD34-PerCP-Cy5.5 (Biolegend, 343612; RRID: AB_2566788), anti-CD45RA-APC-H7 (BD, 560674; RRID: AB_1727497), anti-CD90-PECy7 (BD, 561558; RRID: AB_10714644), anti-CD133-super bright 436 (Ebioscience, 62–1338-42; RRID: AB_2717001), anti-EPCR-PE (Biolegend, 351904; RRID: AB_10900806) and anti-ITGA3-APC (Biolegend, 343808; RRID: AB_10641282). LT-HSCs were defined by the following immunophenotype: CD34^+^CD45RA^−^CD90^+^CD133^+^ITGA3^+^EPCR^+14^. Three microliters of each antibody were used per 1e5 cells in 100μl.

Erythroid differentiation in primary human cells was detected using 2ul each of anti-CD71-PE-Cy7 (Biolegend, 334112; RRID: AB_2563119) and anti-CD235a-APC (Ebioscience, 17–9987-42; RRID: AB_2043823) antibodies in 100ul total volume. Megakaryocyte differentiation was measured using anti-CD41a-PE-Cy7 (BD, 561424; RRID: AB_10642584) and myeloid differentiation was measured using anti-CD14-PE-Cy7 (Biolegend, 367112; RRID: AB_2566714). Erythroid maturation ratio was calculated by dividing the proportion of CD71^+^CD235a^+^ cells by the proportion of CD71^+^CD235a^−^ cells. On day 21 following cell surface staining, enucleation assessment was performed by incubation with Hoechst 33342 stain (Sigma H3570) at 1:10,000 followed by flow cytometry. Percent enucleation was calculated by dividing the proportion of Hoechst negative cells by the proportion of CD71^−^CD235a^+^ cells. Erythroid differentiation of mouse G1E cells was assessed by flow cytometric analysis using anti-mouse Ter119-APC (Ebioscience, 17–5921-82; RRID: AB_469473).

Human cell chimerism after xenotransplantation was determined as we have previously done^25^ by staining with anti-mouse CD45-FITC (Biolegend, 103108; RRID: AB_312973) and anti-human CD45-APC (Biolegend, 368512; RRID: AB_2566372). Human cell subpopulations were detected in the bone marrow of transplanted mice using the following antibodies: anti-human CD45-APC (Biolegend, 368512; RRID: AB_2566372), anti-human CD3-Pacific Blue (Biolegend, 344823; RRID: AB_2563421), anti-human CD19-PECy7 (Biolegend, 302215; RRID: AB_314245), anti-human CD11b-FITC (Biolegend, 301330; RRID: AB_2561703), anti-human CD41a-FITC (Ebioscience, 11–0419-42; RRID: AB_10718234), anti-human CD34-Alexa 488 (Biolegend, 343518; RRID: AB_1937203) and anti-human CD235a-APC (Ebioscience, 17–9987-42; RRID: AB_2043823). Aliquots were stained individually for CD34 and CD235, or with CD45 in conjunction with the other lineage-defining markers. Stem cell number was estimated using ELDA^49^.

GFP was used to track transgene expression in LT-HSCs and other human hematopoietic subpopulations.

Intracellular GATA1 expression was determined by flow cytometry as we have previously done^12^. Briefly, on the indicated days, cells were rinsed with 1% FBS in 1xPBS (GIBCO, 10010–023) and stained for surface markers with anti-CD71-PECy7 (Biolegend, 334112; RRID: AB_2563119) and anti-CD235a-BV421 (BD, 562938; RRID: AB_2721016). Cells were fixed and permeabilized following the manufacturer’s instructions of BD Pharmingen Transcription Factor Buffer Set (562574). 1:100 GATA1 rabbit monoclonal antibody EP2819Y (Abcam, ab76121; RRID: AB_1310256) or 1:200 rabbit monoclonal IgG isotype control (Abcam, ab172730; RRID: AB_2687931) were used as primary antibodies and polyclonal goat anti-rabbit IgG (H+L) Alexa647 conjugate (Jackson, 111–605-003; RRID: AB_2338072) was used as secondary antibody.

Flow cytometric analyses were conducted on Becton Dickinson (BD) LSRII, LSR Fortessa or Accuri C6 instruments and all data were analyzed using FlowJo software (v.10.8). Fluorescence-activated cell sorting (FACS) was performed on BD Aria and samples were collected in PBS containing 2% BSA and 0.01% Tween for immediate processing for sequencing on the 10x Genomics platform.

#### hG1E enhancer construction and viral synthesis

Accessible chromatin upstream of GATA1 was examined in HSCs^20^ and developing erythroid cells^21^. The three enhancer regions (hg19, chrX:48,638,911–48,639,246; chrX:48,641,244–48,641,622; chrX:48,644,267–48,645,053) that make up the hG1E element were synthesized and cloned into pHIV-eGFP (Addgene, 21373) in place of the Ef1a promoter. pHIV-EGFP was a gift from Bryan Welm & Zena Werb^51^. Other vector versions of included 6x binding sites for miR126 or miR223T^27^ or a shorter version of hG1E as shown in Fig. S1E. ChIP-seq data from erythroid cells^52,53^ was analyzed to determine predicted transcription factor binding to hG1E sequences. Clinical-grade hG1E-GATA1 vector was produced by Lentigen Technology, Inc.

To produce research-grade lentivirus, approximately 24 hours prior to transfection, 293T cells were seeded in 10cm plates. Cells were co-transfected with 10μg pΔ8.9, 1μg VSVG, and 10μg hG1E vector variant or shRNA constructs targeting RPS19 or luciferase^12^ using lipofectamine 3000 (Thermo, L3000001). Viral supernatant was harvested at 48 hours post-transfection, filtered with a 0.45um filter and concentrated by ultracentrifugation at 100,000 × g for 2 hours at 4°C.

Viral transduction was performed in HSC media on day 1 following thawing of CD34^+^ cells or day 1 following CD34 selection of bone marrow MNCs. In the RPS19 CRISPR experiments, cells were edited on day 1 and infected on day 2. For the shRNA experiments, cells were co-infected with hG1E construct and shRNA virus or controls. 1–3e5 CD34^+^ cells or 1–3e6 bone marrow MNCs were transduced at a multiplicity of infection (MOI) of 10, in 12 well plates with 8μg/ml of polybrene (Millipore, TR-1003-G), spun at 931 × g for 1.5 hours at 21°C and incubated in the viral supernatant overnight at 37°C. Virus was washed off 16 hours after infection and cells were transferred to Phase I erythroid media at 48 hours after transduction.

#### CRISPR editing and analysis

Electroporation was performed on day 1 after thawing HSPCs using the Lonza 4D Nucleofector with 20 μl Nucleocuvette strips as described^25^. Conditions were titrated to achieve editing of 50% of alleles. Briefly, ribonucleoprotein (RNP) complex was made by combining 50pmol Cas9 (IDT) and 50pmol modified sgRNA (Synthego) targeting RPS19 (5’- ACGUCUUUUACAGUAACUCC-3’) or AAVS1 (5’-GGGGCCACUAGGGACAGGAU-3’) and incubating at 21°C for 15 minutes. 2e5–4e5 HSPCs were resuspended in 20 μl P3 solution were mixed with RNP and underwent nucleofection with program DZ-100. Cells were returned to HSC media and editing efficiency was measured by PCR at 72 hours after electroporation, at the indicated time during erythroid differentiation or of individual BFU-E at 12 days. First, genomic DNA was extracted using the DNeasy kit (Qiagen) according to the manufacturer’s instructions. Genomic PCR was performed using Platinum II Hotstart Mastermix (Thermo) and edited allele frequency was detected by Sanger sequencing and was analyzed by ICE (ice.syngthego.com). The following primer pairs were used: RPS19 (forward: 5’- TTTAGGATGCGCTGGAGCGA-3’; reverse: 5’- CACAACTATGCTGTGCCCAG-3’).

#### Vector copy number

To determine vector copy number, cells were transduced with hG1E-GATA1 vector, and post-transduction sample was collected from bulk HSPCs on day 7 of HSC culture. Erythroid progenitor samples were collected on day 18 of erythroid culture. *In vivo* samples were collected from human CD45-selected cells harvested from xenotransplant recipient bone marrow at 16 weeks after transplantation. Vector copy number (VCN) was determined by duplex quantitative polymerase-chain-reaction (qPCR) for the detection of proviral DNA copy numbers per host cell genome using the Biotec MACS COPYcheck kit (Miltenyi, 130–128-157) according to manufacturer’s recommendations, as previously described^54^.

#### Quantitative real time PCR (qRT-PCR)

Total mRNA was harvested from the indicated hematopoietic cell populations after FACS, using Qiagen RNeasy according to the manufacturer’s recommendations (Qiagen, 74004), followed by cDNA synthesis using the iScript cDNA Synthesis kit (Biorad, 1708841). *GATA1* expression was quantified by qPCR on CFX96 Real-time PCR detection system (Biorad) by measuring SYBR Green (Biorad, 74004) incorporation. *GATA1* expression levels in each hematopoietic subpopulation were normalized to the bulk human cell population.

### QUANTIFICATION AND STATISTICAL ANALYSIS

Statistical tests and statistical significance are indicated in the figure legends. All error bars represent standard error of the mean unless otherwise indicated.

#### Single cell RNA sequencing

CD34^+^ cells from patients BCH-002 and BCH-008 were treated with hG1E-GATA1 or HMD-GFP and cultured for 10 days in erythroid differentiation media prior to collection and immediate loading onto four lanes of 10x RNA 3’ V3 kit (10x Genomics, PN-1000269) according to the manufacturer’s guidelines. Libraries were constructed with distinct i7 barcodes, pooled in equal molecular concentrations and pooled 4nM barcoded gene expression libraries containing 1% PhiX Control v3 (Illumina, FC-110–3001) were sequenced with NovaSeq 6000 S1 reagents (Illumina) in a pair-ended mode with 28bp for read1, 10bp for index1, 90bp for read2, 10bp for index2. The following number of cells and reads per cell were recovered for each sample BCH-002 GFP: 11,445 cells, 38,779 reads per cell; BCH-002 hG1E: 9,859 cells, 53,748 reads per cell; BCH-008 GFP: 1,007 cell, 476,388 reads per cell; BCH-008 hG1E: 6,659 cells, 63,883 reads per cell. Demultiplexed reads with BCL convert v3.10.5 (Illumina) were then aligned to hg38 version of the human genome, implemented with the sequence of GFP and codon-optimized exogenous GATA1 (hG1E-GATA1) using Cell Ranger v7.2.0 (10x Genomics). Output files were then processed with Seurat package v5.0.1^55^ to build an rds object. After filtering each sample for nFeature_RNA > 300 & < 9000 & percentage of mitochondrial genes < 15%, 23,424 cells were analyzed. Data were centered and scaled for all genes using a linear regression model. Dimensionality reduction was performed with a principal component analysis (PCA) on the top 2,000 variable features, and clusters were determined based on the top 30 principal components identified by an elbow plot to run the UMAP. Clusters were manually annotated based on the expression of top 10 gene markers and controlled by projection onto a published bone marrow dataset^56^. Transgenic cells were identified by calculating RNA counts of hG1E-GATA1 normalized to the total RNA counts of the cell. Pseudotime analysis was performed with Monocle3 package^57^. For a better comparison to existing literature, Gene Set Enrichment Analysis (GSEA) was performed with the Hallmark and KEGG gene sets from MSigDB collection. Specific GATA1 signature was browsed in the CGP sub-collection of MSigDB.

#### Integration site analysis

Genomic DNA was collected from the indicated samples and viral integration events were identified by ligation-mediated PCR after random fragmentation followed by next generation sequencing and analysis with the INSPIIRED pipeline as previously described^39^.

Two hyperabundant sequences were removed from the integration site sequence analysis. One corresponded to the codon-optimized GATA1 transgene, and thus was vector-derived. The second sequence annotated as *TBC1D5* was present in multiple samples from independent runs, but not negative controls, and has 100% sequence identity to many lentiviral vectors and was presumed to be a vector-derived sequence. The full vector sequence of our clinical grade lentivirus was not available for comparison. The unfiltered integration sites are listed in Table S2.

The analysis comparing the gene targeting rates among datasets and clone growth analysis was performed using the MELISSA R package^58^.

Briefly, to estimate gene-specific integration site rates, MELISSA used a logistic regression model to identify over/under-targeted genes and calculate gene-specific targeting rates. The modeling approach considered the presence/absence of Integration Site (IS) at each genomic coordinate as a binary variable (1 if an IS maps to the specific location and 0 otherwise) and measured the probability of an IS event occurring in a particular gene compared to the remaining part of the genome. The two-conditions differential analysis was specifically designed to allow IS rates to vary across genes and detect only genes with an unshared variation of the gene-targeting rates between our experimental data and a reference IS datasets generated using CD34^+^ HSPC isolated from a healthy donor mobilized peripheral blood transduced with EGFP lentiviral vector^40^.

Statistical testing was performed on the gene IS-enrichment score, measuring the ratio between the gene’s IS rate and the baseline, sample-specific, genome-wide IS rate. This model allowed intrinsically normalizing datasets for factors such as gene length and the number of IS retrieved. The analysis returned a table containing a list of all the targeted genes and their related gene-targeting scores as an output. These scores were used to generate the genome-wide Miami plots and the high-risk genes^59^ waterfall plots.

In all the scenarios described, we tested the association of genes using a set of Likelihood Ratio Test (LRT), and P-values were then adjusted using the False Discovery Rate (FDR) method (q-value<0.05). The gene targeting score corresponded to gene-specific LRT statistic, multiplied by the sign of the estimated parameter in case of a two groups analysis.

#### Data reporting

No statistical methods were used to pre-determine sample sizes but our sample sizes were similar to those reported in previous publications^14,21,^. Data distribution was assumed to be normal, but this was not formally tested. Data collection and analysis were not performed blind to the conditions of the experiments. No animals or data points were excluded from analysis.

## Supplementary Material

1 **Document S1:** Figures S1–S7. Table S1.

2 **Table S2: Integration sites of hG1E-GATA1 vector, related to** Figure 7 and Figure S7. Integration sites of hG1E-GATA1 in LT-HSCs compared to bulk HSCs and in erythroid progenitors compared to bulk HSCs are shown.

## Figures and Tables

**Key resources table:** 

REAGENT or RESOURCE	SOURCE	IDENTIFIER
Antibodies		
anti-human CD34-PerCP-Cy5.5 (clone: 561)	Biolegend	Cat: 343612; RRID: AB_2566788
anti-human CD34-Alexa 488 (clone: 581)	Biolegend	Cat: 343518; RRID: AB_1937203
anti-human CD45RA-APC-H7 (clone: HI100)	BD	Cat: 560674; RRID: AB_1727497
anti-human CD90-PECy7 (clone: 5E10)	BD	Cat: 561558; RRID: AB_10714644
anti-human CD133-super bright 436 (clone: TMP4)	Ebioscience	Cat: 62-1338-42; RRID: AB_2717001
anti-human EPCR-PE (clone: RCR-401)	Biolegend	Cat: 351904; RRID: AB_10900806
anti-human ITGA3-APC (clone: ASC-1)	Biolegend	Cat: 343808; RRID: AB_10641282
anti-human CD71-PE-Cy7 (clone: CY1G4)	Biolegend	Cat: 334112; RRID: AB_2563119
anti-human CD235a-APC (clone: HIR2)	Ebioscience	Cat: 17-9987-42; RRID: AB_2043823
anti-human CD235a-BV421 (clone: HIR2)	BD	Cat: 562938; RRID: AB_2721016
anti-human CD41a-PE-Cy7 (clone: HIP8)	BD	Cat: 561424; RRID: AB_10642584
anti-human CD41a-FITC (clone: HIP8)	Ebioscience	Cat: 11-0419-42; RRID: AB_10718234
anti-human CD14-PE-Cy7 (clone: 63D3)	Biolegend	Cat: 367112; RRID: AB_2566714
anti-human CD45-APC (clone: 2D1)	Biolegend	Cat: 368512; RRID: AB_2566372
anti-human CD3-Pacific Blue (clone: SK7)	Biolegend	Cat: 344823; RRID: AB_2563421
anti-human CD19-PECy7 (clone: HIB19)	Biolegend	Cat: 302215; RRID: AB_314245
anti-human CD11b-FITC (clone: ICRF44)	Biolegend	Cat: 301330; RRID: AB_2561703
anti-human GATA1 (clone: EP2819Y)	Abcam	Cat: ab76121; RRID: AB_1310256
anti-human IgG (clone: EPR25A)	Abcam	Cat: ab172730; RRID: AB_2687931
anti-mouse IgG-Alexa647 (clone: polyclonal)	Jackson	Cat: 111-605-003; RRID: AB_2338072
anti-mouse Ter119-APC (clone: TER-119)	Ebioscience	Cat: 17-5921-82; RRID: AB_469473
anti-mouse CD45-FITC (clone: 30-F11)	Biolegend	Cat: 103108; RRID: AB_312973
Hoechst 33342	Sigma-Aldrich	Cat: H3570
Bacterial and virus strains		
OneShot TOP10 Chemically Competent Cells	Invitrogen	Cat: C404006
hG1E-GATA1	This study	Lentigen Technology, Inc
hG1E-GATA1-IRES-GFP	This study	Lentigen Technology, Inc
		
		
Biological samples		
Human CD34^+^ hematopoietic stem and progenitor cells, adult	Fred Hutchinson Cancer Research Center	N/A
Cord Blood Unit for umbilical cord-derived CD34^+^ hematopoietic stem and progenitor cells	Dana Farber Pasquarello Tissue Bank	N/A
Bone marrow mononuclear cells from DBA patients	Research sample banking repositories at Boston Children’s Hospital, University of Cincinnati College of Medicine, and University of Colorado Anschutz Medical Campus	N/A
		
		
Chemicals, peptides, and recombinant proteins		
Dulbecco’s Modified Eagle Medium-High Glucose (DMEM)	Life Technologies	Cat: 11965-118
Iscove’s Modified Dulbecco’s Medium (IMDM)	Life Technologies	Cat: 12440-061
Ficoll-Paque		
StemSpan SFEM II medium	Stem Cell Technologies	Cat: 09605
StemSpan CC100	Stem Cell Technologies	Cat: 02690
Recombinant Human Thrombopoietin	Peprotech	Cat: 300-18
UM171	Stem Cell Technologies	Cat: 72912
Human Serum, Type AB	Atlanta Biologicals	Cat: S40110
Human Plasma, Type AB	SeraCare	Cat: 1810-0001
Penicillin-Streptomycin	Life Technologies	Cat: 15140-122
Humulin R (Insulin)	Lilly	Cat: NDC 0002-8215-01
Heparin	Hospira	Cat: NDC 00409-2720-01
Human holo-transferrin	Sigma-Aldrich	Cat: T0665
Recombinant human stem cell factor (SCF)	Peprotech	Cat: 300-07
Recombinant mouse stem cell factor (SCF)	Peprotech	Cat: 250-03
Epogen (recombinant erythropoietin)	Amgen	Cat: NDC 55513-267-10
Recombinant human interleukin-3 (IL3)	Peprotech	Cat: 200-03
Fetal Bovine Serum (FBS)	BioTechne	Cat: S11550
L-Glutamine	Thermo Fisher Scientific	Cat: 25-030-081
1-thioglycerol	Sigma-Aldrich	Cat: M6145
PBS	GIBCO	Cat: 10010-023
Lipofectamine 3000	Thermo Fisher Scientific	Cat: L3000001
Polybrene Infection/Transfection reagent	Millipore	Cat: TR-1003-G
PhiX Control v3	Illumina	Cat: FC-110-3001
		
		
		
		
		
		
Critical commercial assays		
EasySep Human CD34 Positive Selection Kit II	Stem Cell Technologies	Cat: 17896
MethoCult H4434	Stem Cell Technologies	Cat: 04434
Pharmingen Transcription Factor Buffer Set	BD	Cat: 562574
Biotec MACS COPYcheck	Miltenyi	Cat: 130-128-157
10x RNA 3’ V3 kit	10x Genomics	Cat: PN-10000269
NovaSeq 6000 S1	Illumina	Cat: 20028318
		
Deposited data		
scRNA sequencing of DBA patient samples treated with hG1E-GATA1	This study	GSE261450
		
		
		
		
Experimental models: Cell lines		
G1E	Khajuria et al^12^	Doi: 10.1016/j.cell.2018.02.036;
293T cells	ATCC	Cat: CRL-3216
		
		
		
Experimental models: Organisms/strains		
Mouse:* Cg-Kit^W-41J^Tyr^+^Prkdc^scid^Il2rg^tm1Wjl^*/ThomJ (NBSGW)	Jackson Laboratory	RRID: IMSR_JAX:026622
		
		
		
		
		
Oligonucleotides		
sgRPS19: ACGUCUUUUACAGUAACUCC	This study	N/A
sgAAVS1: GGGGCCACUAGGGACAGGAU	Voit et al^25^	Doi: 10.1038/s41590-022-01370-4
RPS19_F: TTTAGGATGCGCTGGAGCGA	This study	N/A
RPS19_R: CACAACTATGCTGTGCCCAG	This study	N/A
		
Recombinant DNA		
		
		
		
		
		
Software and algorithms		
Flowjo v10.8	N/A	N/A
BCL Convert v3.10.5	Illumina	N/A
CellRanger v7.2.0	10x Genomics	N/A
Seurat v5.0.1	Hao et al^55^	Doi: 10.1038/s41587-023-01767-y
Monocle3	Qiu et al^57^	Doi: 10.1038/nmeth.4402
INSPIIRED	Sherman et al^39^	Doi: 10.1016/j.omtm.2016.11.002
MELISSA R package	Kapourani et al^58^	Doi: 10.1186/s13059-019-1665-8
Other		
		
		
		
		
		

## Highlights

- Mutations in dozens of genes cause DBA constraining traditional gene therapy approaches
- DBA mutations converge on impaired translation of GATA1
- Erythroid-restricted expression of GATA1 increases RBC output in DBA patient samples
- Clinical-grade lentiviral vector causes no impaired HSC function or clonal expansion
