# Supplementary material for: Regulated GATA1 expression as a universal gene therapy for Diamond-Blackfan anemia
Source: Cell Stem Cell. Author manuscript; Available in PMC 2025 Jan 4. (PMC11698655; doi:10.1016/j.stem.2024.10.012)
Supplement: 1 — Document S1: Figures S1–S7. Table S1. [file NIHMS2031095-supplement-1.pdf]

Figure S1. Optimization of lineage-restricted expression, related to Figure 1

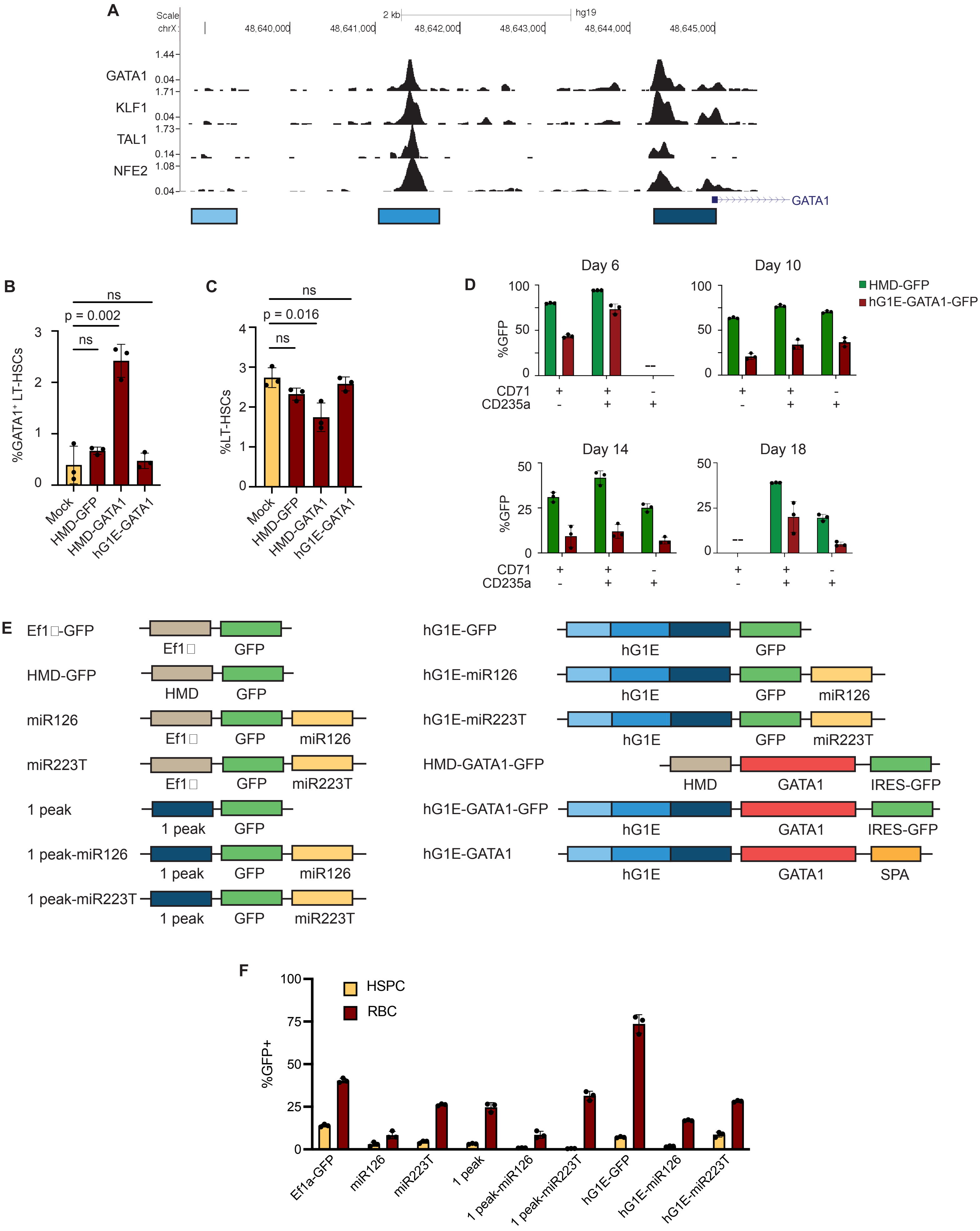

Figure S2. Preserved stem cell function after hG1E-GATA1 treatment, related to Figure 2

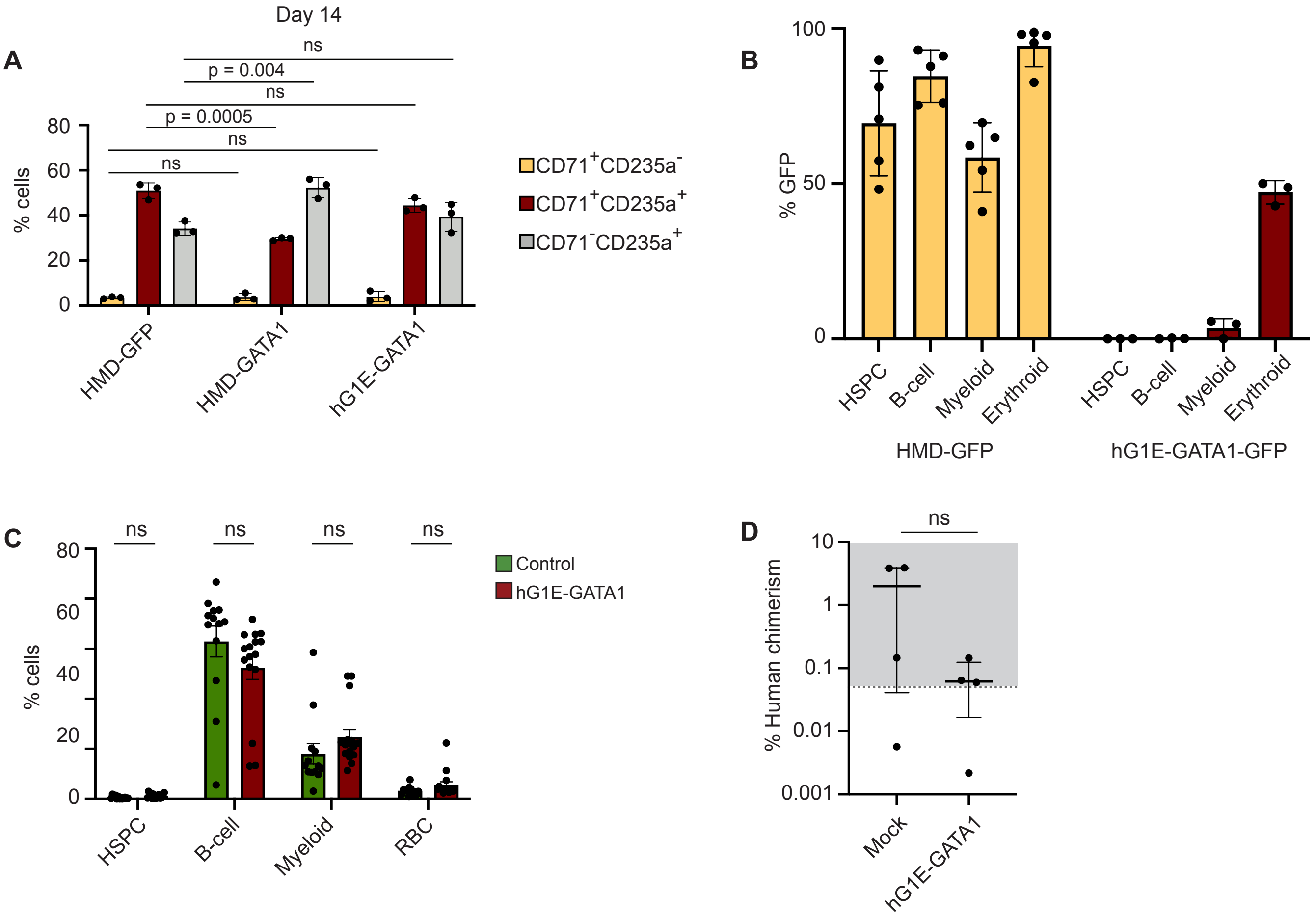

Figure S3. Generation of a CRISPR model of DBA, related to Figure 3

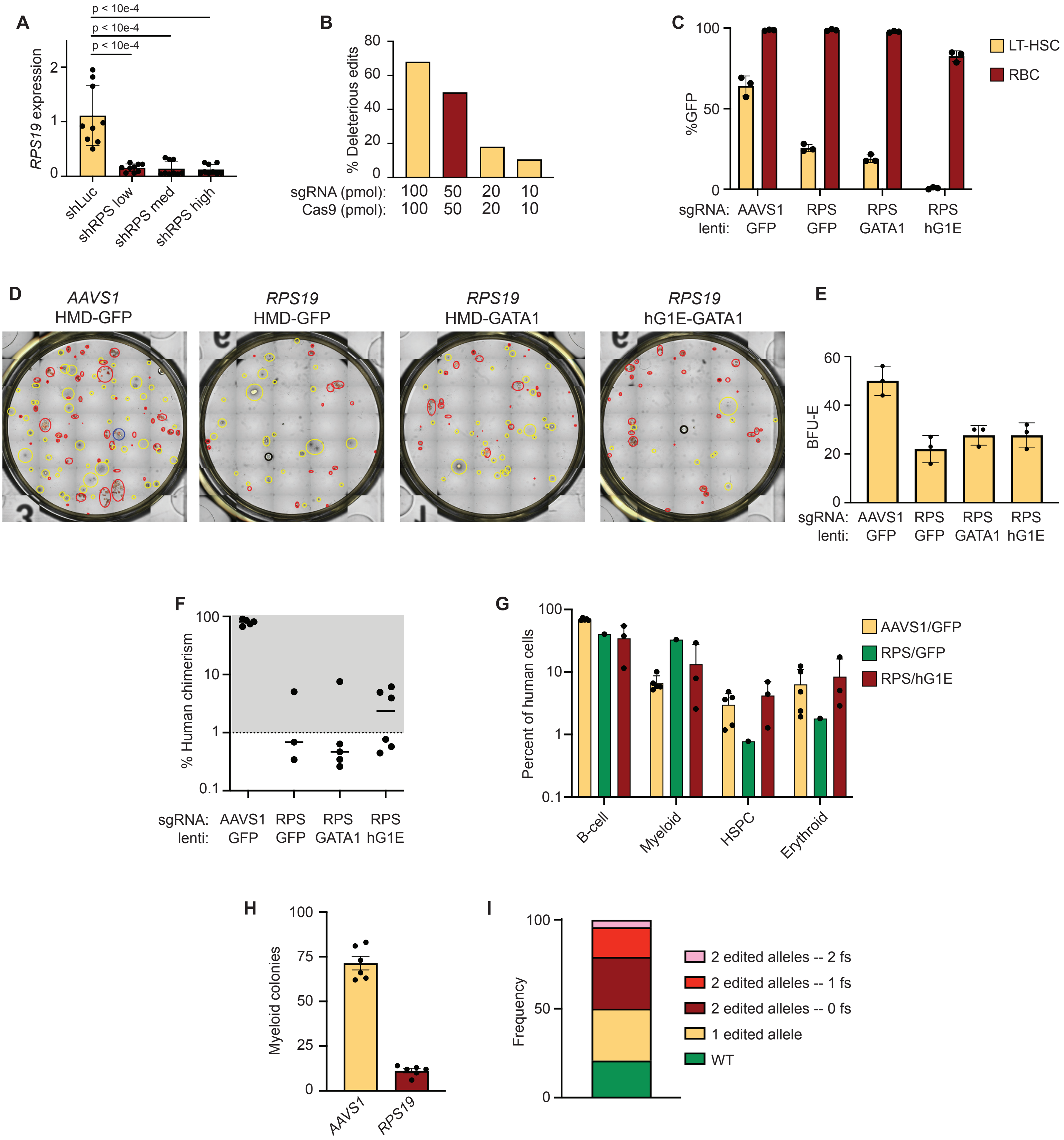

Figure S4. Increased erythroid output in DBA patients across genotypes, related to Figure 4

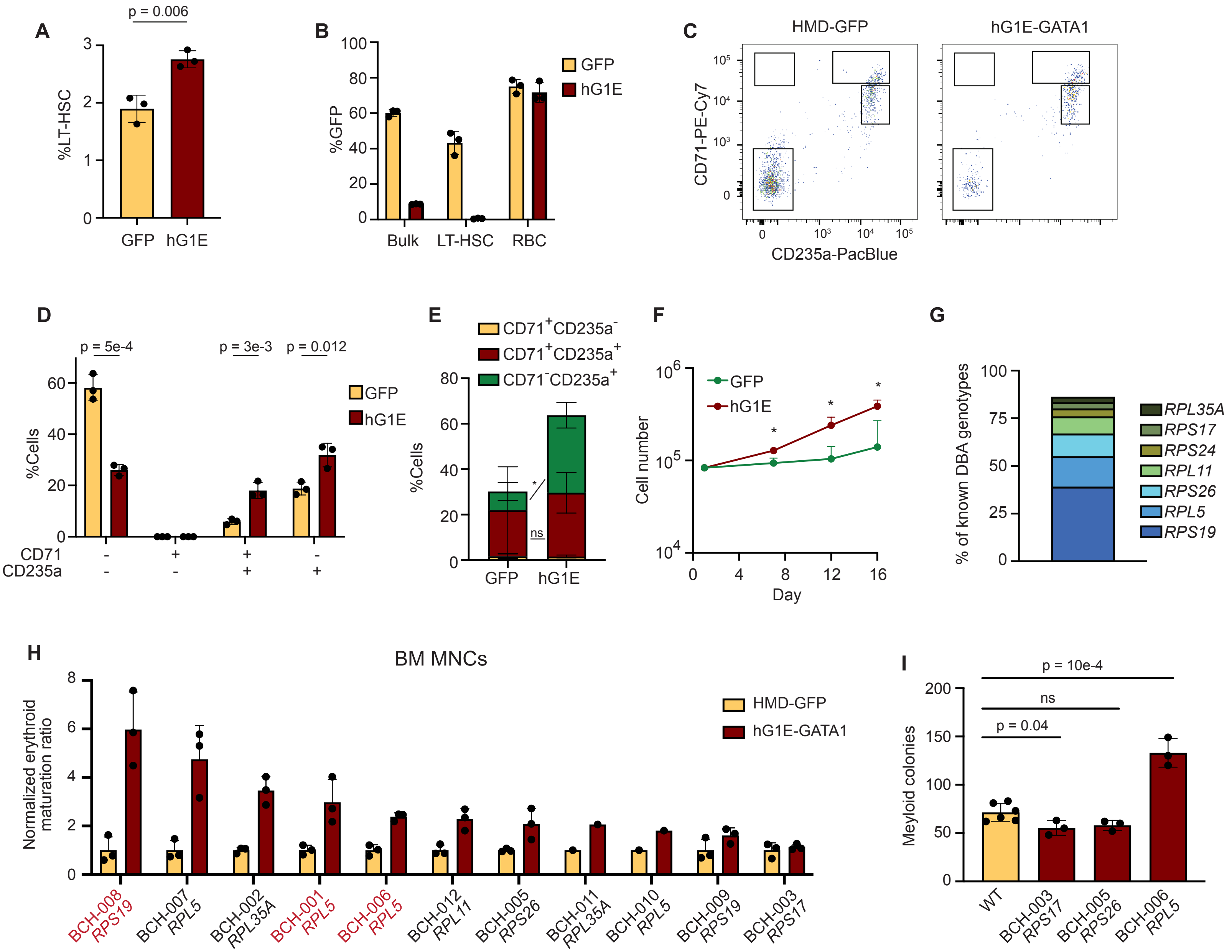

Figure S5. hG1E-GATA1 stimulates erythroid output in DBA samples *in vivo*, related to Figure 5

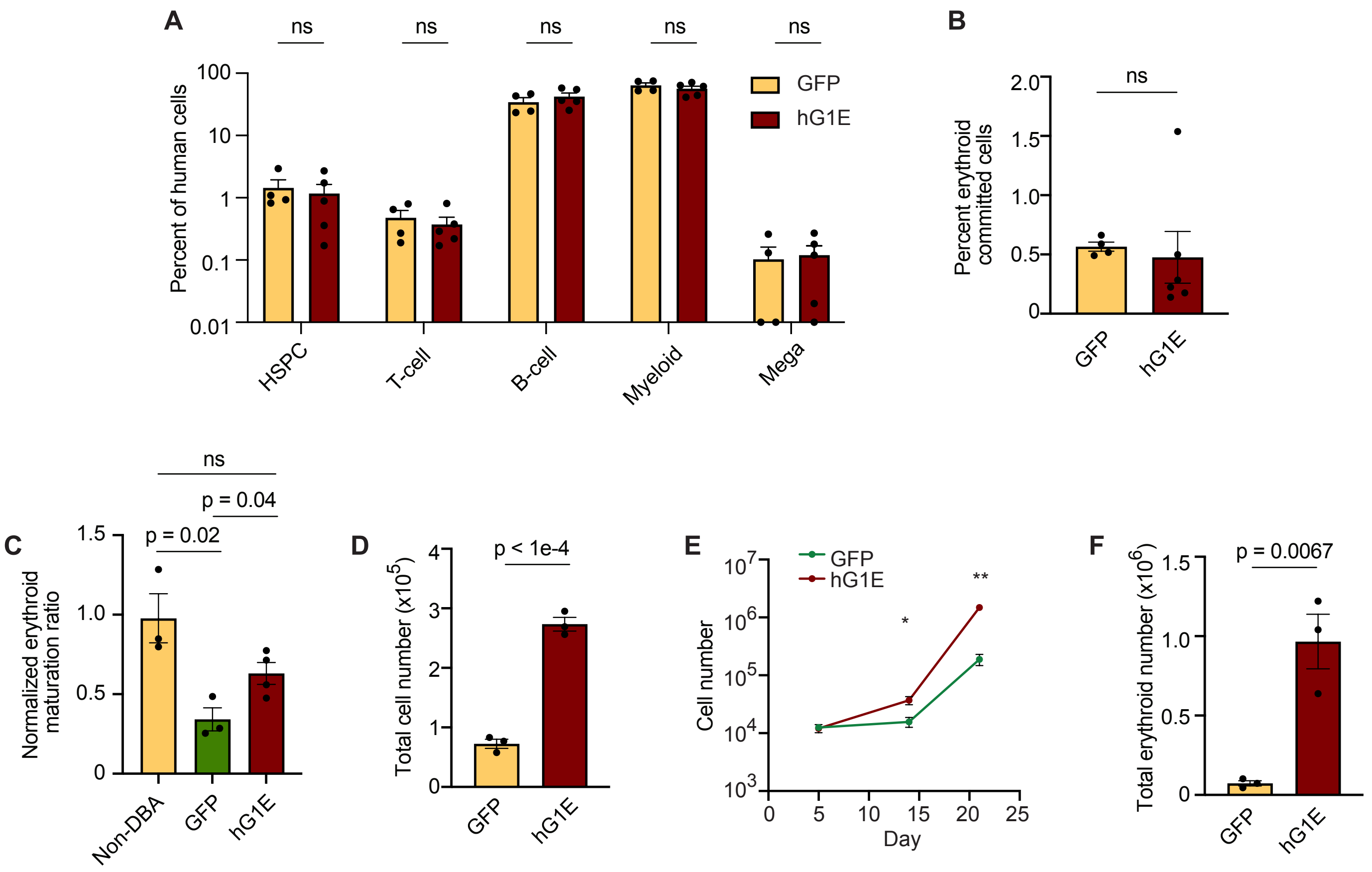

Figure S6. Transcriptional effects of hG1E-GATA1 therapy, related to Figure 6

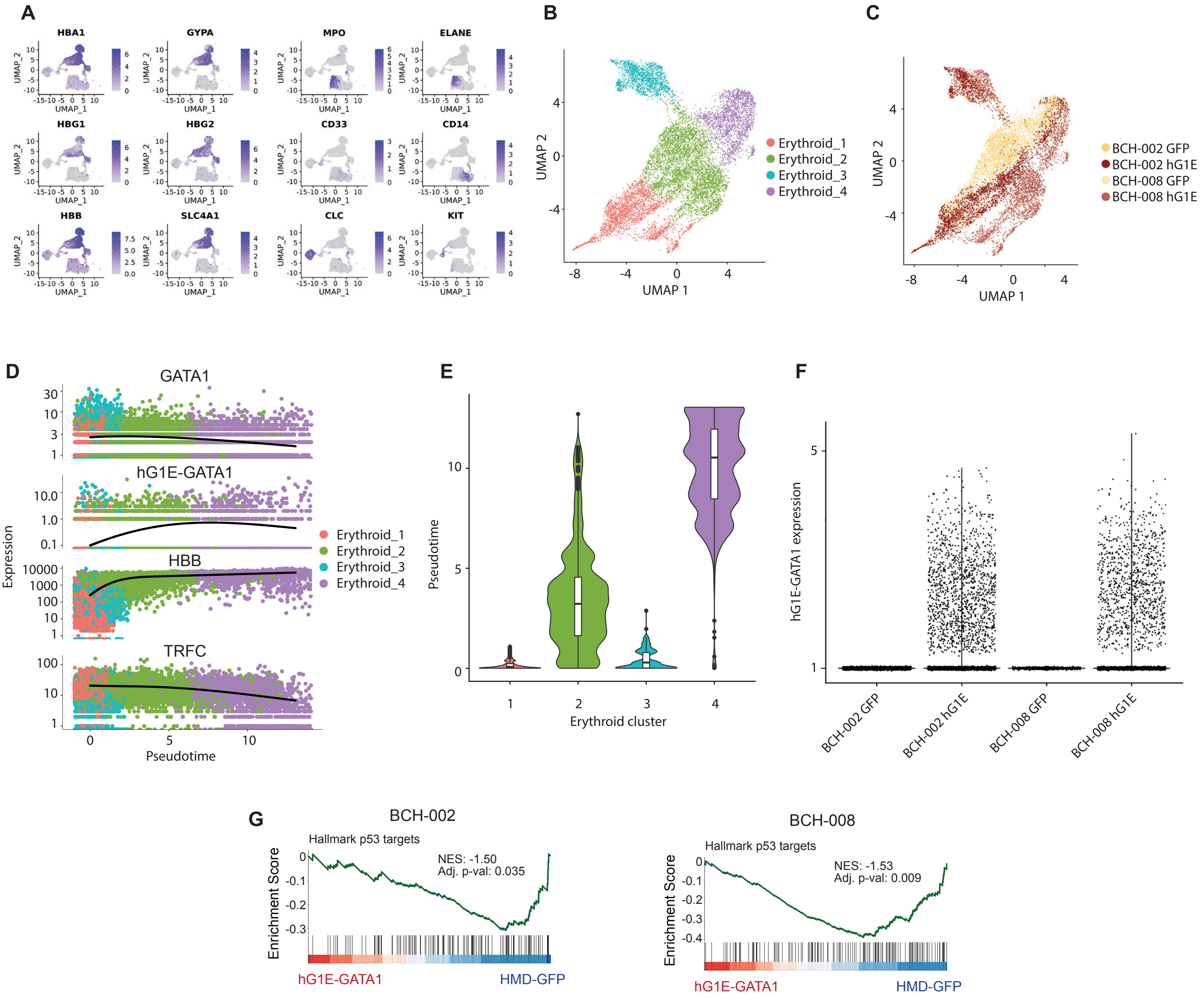

Figure S7. Safe genomic integration of hG1E-GATA1, related to Figure 7

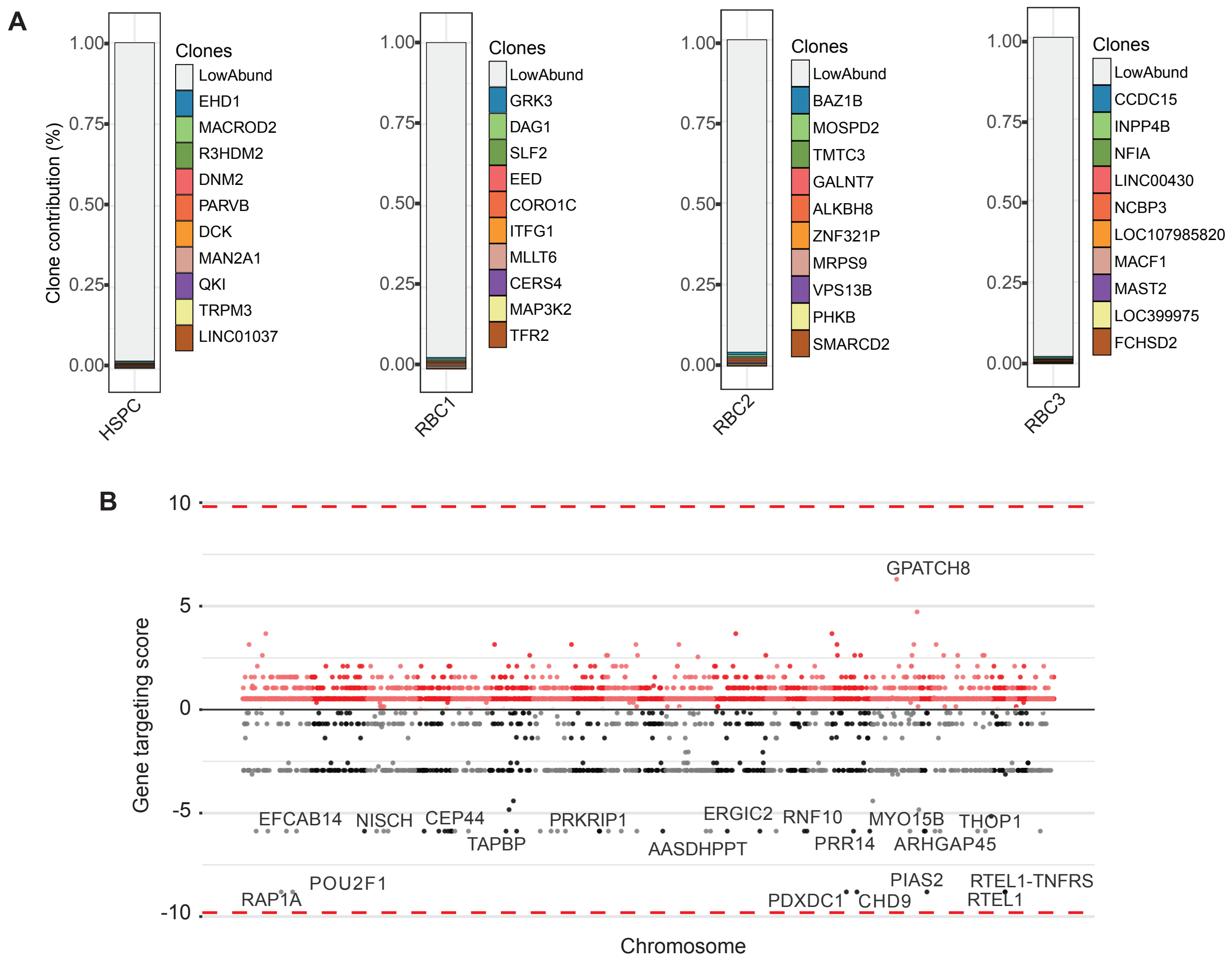

Patient ID	Affected gene	Mutation	AA change	Clinical status
BCH-001	*RPL5*	c.173 del	p.R58K fs*12	Transfusion dependent
BCH-002	*RPL35A*	chr3:1197951314 A>T	Splice donor	Transfusion dependent
BCH-003	*RPS17*	deletion of chr15q25.2	deletion	Transfusion dependent
BCH-004	*RPS24*	c.20 T>G	p.I7S	Transfusion dependent
BCH-005	*RPS26*	c.230G>A	p.C77Y	Transfusion dependent
BCH-006	*RPL5*	c.222dupA	p.V75S fs*38	Remission
BCH-007	*RPL5*	c.169_172delAACA	p.N57E fs*12	Transfusion dependent
BCH-008	*RPS19*	c.184C>T	p.R62W	Transfusion dependent
BCH-009	*RPS19*	c.185G>A	p.R62Q	Transfusion dependent
BCH-010	*RPL5*	c.535C>T	p.R179*	Remission
BCH-011	*RPL35A*	c.82_84delCTT	p.L28del	Transfusion dependent
BCH-012	*RPL11*	c.94_97delAGAC	p.R32*	Remission

Table S1. Genotypes and clinical status of patient samples used in this study, related to Figure 4 and Figure S4

Supplemental Figure Legends

Figure S1. Optimization of lineage-restricted expression of hG1E, related to Figure 1.

A. Chromatin occupancy of erythroid transcription factors in primary human erythroid progenitors [S1, S2]. Blue bars below the genome browser represent the putative enhancer elements that make up the hG1E sequence.

B. Evaluation of GATA1 protein expression in immunophenotypic LT-HSCs. On day 4 following transduction, cells were stained for surface expression of CD34, CD45RA, CD90, CD133, and EPCR, before fixation, permeabilization, and staining for intracellular GATA1. Displayed is the percentage of LT-HSCs expressing GATA1. n = 3 independent replicates. Mean, S.E.M, and P-value are shown. ns – not significant.

C. Evaluation of immunophenotypic LT-HSCs. Using the FACS gating strategy shown in **Fig. 1D**, the percentage of LT-HSCs was determined on day 4 after treatment with the indicated vector. n = 3 independent replicates. Mean, S.E.M, and P-value are shown. ns – not significant.

D. GFP expression during erythroid differentiation. Following transduction of primary human HSPCs with the indicated vector, cells were cultured in erythroid differentiation media and percent GFP positive cells was determined by flow cytometry on the indicated days. Percentage of cells expressing GFP in each subpopulation defined by the markers along the x-axis is shown. n = 3 independent replicates. Mean and S.E.M are shown.

E. Lentiviral vectors used in this study. Ef1a – elongation factor 1 alpha promoter, HMD – HIV/MSCV hybrid LTR promoter, miR126 – binding site for microRNA 126, miR223T – binding site for microRNA 223T, 1 peak – proximal ATAC peak of GATA1 locus, hG1E – human GATA1 enhancer, IRES – internal ribosome entry site, SPA – synthetic polyadenylation sequence.

F. Comparison of GFP expression in HSPCs and erythroid cells. Cells were transduced with the indicated vector and cultured in HSC media or erythroid differentiation media. Percentage of cells expressing GFP in the bulk HSPC population on day 7, and in the $CD71^{+}CD235a^{+}$ erythroid progenitor population are shown. n = 3 independent replicates. Mean and S.E.M are shown.

Figure S2. Preserved stem cell function after hG1E-GATA1 treatment, related to Figure 2.

A. Evaluation of erythroid differentiation after hG1E-GATA1-GFP treatment. On day 14 of erythroid culture, the stage of differentiation was assessed by flow cytometry in samples treated with HMD-GFP, HMD-GATA1, or hG1E-GATA1. The percentage of early erythroid progenitors expressing CD71 (yellow), and more mature progenitors expressing both CD71 and CD235a (red), or CD235a only (gray) are shown. n = 3 independent replicates, mean and S.E.M are shown. Two-sided Student *t*-test was used for comparisons. P values are shown. ns – not significant.

B. Lineage-restricted expression from hG1E-GATA1-GFP *in vivo*. Human HSPCs transduced with HMD-GFP or hG1E-GATA1-GFP were transplanted into NBSGW mice and bone marrow samples were harvested at 16 weeks. GFP expression was analyzed by flow cytometry in human hematopoietic subpopulations as defined by the following markers: HSPC, $CD34^{+}$, B-cell, $CD45^{+}CD19^{+}$; myeloid, $CD45^{+}CD33^{+}$; erythroid, $CD71^{+}$.

C. Human hematopoietic lineages after xenotransplantation. 16 weeks after xenotransplantation, bone marrow harvest from recipient mice was performed. Human hematopoietic lineages were determined using flow cytometry for the following lineage-defining markers: HSPC, $CD34^+$, B-cell, $CD45^+CD19^+$; myeloid, $CD45^+CD33^+$; RBC, $CD235a^+$. Each marker represents a recipient mouse with human chimerism >1%. Mean and S.E.M are shown.

D. Evaluation of human chimerism after secondary xenotransplantation. 2e5 $CD34^+$ cells from primary xenotransplant recipients were transplanted by tail vein injection and chimerism was assessed in the bone marrow at 16 weeks. Human chimerism was determined by comparing the percentage of human $CD45^+$ cells to the percentage of mouse $CD45^+$ cells by flow cytometry. Each marker represents one recipient mouse. Median with interquartile range is shown. Two-sided Student *t*-test was used for comparisons. ns – not significant.

Figure S3. Generation of a CRISPR model of DBA, related to Figure 3.

A. Knockdown of *RPS19* expression by shRNA. Human $CD34^+$ cells were transduced with the indicated shRNA lentivirus at an MOI of 1 (low), 3 (med), or 10 (high) before puromycin selection and culture in erythroid differentiation media for 6 days. *RPS19* expression was measured by qPCR and normalized to control. n = 9 independent replicates, mean and S.E.M are shown. Two-sided Student *t*-test was used for comparisons. P values are shown.

B. Optimization of CRISPR conditions to achieve *RPS19* haploinsufficiency. RNPs were generated using the indicated amounts of Cas9 and sgRNA and nucleofected into primary human HSPCs. Deleterious edits as defined in **Fig. 3D** were quantified on day 7 of HSC culture by PCR and Sanger sequencing.

C. Bar plot of GFP expression in *RPS19* edited cells. Following the experimental outline in **Fig. 3E**, the percentage of GFP positive LT-HSCs and $CD71^+CD235a^+$ erythroid progenitors was determined by flow cytometry on day 6 of HSC culture or day 8 or erythroid culture. RPS: RPS19, GFP: HMD-GFP, GATA1: HMD-GATA1, hG1E: hG1E-GATA1-IRES-GFP. Mean and S.E.M are shown.

D. Erythroid colony formation after CRISPR editing and viral transduction. Primary human HSPCs were treated with the indicated CRISPR RNP and vector and plated in methylcellulose as described in **Fig. 3G**. Colonies were imaged and identified using StemVision with manual verification. Red circles indicate burst forming unit – erythroid (BFU-E) colonies and yellow circles indicate colony forming unit – granulocyte (CFU-G) or – macrophage (CFU-M). Each picture is representative of three independent replicates.

E. Quantification of erythroid colonies. BFU-E colonies from **Fig. S3D** were counted in each sample treated with the indicated sgRNA RNP and lentivirus. RPS: RPS19, GFP: HMD-GFP, GATA1: HMD-GATA1, hG1E: hG1E-GATA1-IRES-GFP. n = 3 independent replicates, mean and S.E.M are shown.

F. Evaluation of human chimerism. 16 weeks after xenotransplantation, bone marrow harvest from recipient mice was performed. Human chimerism was determined by comparing the percentage of human $CD45^+$ cells to the percentage of mouse $CD45^+$ cells by flow cytometry. Each marker represents one recipient mouse. Mice with human chimerism >1% were used in subsequent analyses. RPS: RPS19, GFP: HMD-GFP, GATA1: HMD-GATA1, hG1E: hG1E-GATA1-IRES-GFP.

G. Human hematopoietic lineages after xenotransplantation. Lineages were determined using flow cytometry for the following lineage-defining markers: B-cell, $CD45^+CD19^+$; myeloid, $CD45^+CD33^{+,}$ HSPC, $CD34^+$; erythroid, $CD235a^+$. Each marker represents a recipient mouse with human chimerism >1%. AAVS1: AAVS1 sgRNA, RPS: RPS19 sgRNA, GFP: HMD-GFP, GATA1: HMD-GATA1, hG1E: hG1E-GATA1-IRES-GFP. Mean and S.E.M are shown.

H. Quantification of myeloid colonies. Human $CD34^+$ HSPCs from healthy donors were treated with either *AAVS1* or *RPS19* RNP and plated in methylcellulose. CFU-G, CFU-M, and CFU-GM colonies were counted on day 12. Colonies were imaged and identified using StemVision with manual verification. n = 6 independent replicates, mean and S.E.M are shown.

I. Genotyping of myeloid colonies. Genomic DNA was collected from individual CFU-G, CFU-M, and CFU-GM colonies from the *RPS19* edited samples from **Fig. S3H** on day 12. *RPS19* genotyping was performed by PCR amplification and Sanger sequencing. Frequency of the indicated editing outcomes including total edited alleles and frameshift (fs) edits. Number of genotyped colonies: 24.

Figure S4. Increased erythroid output in DBA patients across genotypes, related to Figure 4.

A. Evaluation of immunophenotypic LT-HSCs from DBA patient BCH-001. Using the FACS gating strategy shown in **Fig. 1D**, the percentage of LT-HSCs was determined on day 6 in HSC culture. GFP: HMD-GFP, hG1E: hG1E-GATA1-IRES-GFP n = 3 independent replicates. Mean, S.E.M, and P-value are shown.

B. Lineage-restricted expression of GFP in DBA patient sample. CD34-selected HSPCs from DBA patient BCH-001 were treated with the indicated vector and cultured in HSC media or erythroid differentiation media. Percentage of cells expressing GFP was determined for bulk HSPC population ("Bulk") and LT-HSC population on day 6 of HSC culture and for $CD71^+CD235a^+$ erythroid progenitors on day 8 of erythroid culture. GFP: HMD-GFP, hG1E: hG1E-GATA1-IRES-GFP. n = 3 independent replicates. Mean and S.E.M are shown.

C. Representative flow cytometry plots of erythroid differentiation of DBA patient sample. On day 11 of erythroid culture, BCH-001 patient samples treated with the indicated vectors were analyzed by flow cytometry. Boxes shown correspond to the populations plotted in **Fig. S4D**.

D. Quantification of erythroid progenitor populations. Subpopulation distribution of cells from DBA patient BCH-001 on day 11 of erythroid culture are shown. n = 3 independent replicates. Mean, S.E.M, and P-value are shown.

E. Quantification of erythroid subpopulations. Cells from patient BCH-006 undergoing *in vitro* erythroid differentiation were analyzed by flow cytometry for expression of CD71 and CD235a on day 14. Mean and S.E.M are shown. Two-sided Student *t*-test was used for comparisons. * $P < 0.005$. ns, not significant.

F. Total cell number during erythroid differentiation. HSPCs from DBA patient BCH-001 were treated with the indicated vectors and cultured in erythroid differentiation media. Cell number was quantified using Trypan blue exclusion on the indicated days. Mean and S.E.M. are shown. Absent error bars are obscured by the size of the markers. Two-sided Student *t*-test was used for comparisons. * $P < 0.05$.

G. Efficacy of hG1E-GATA1 across DBA genotypes. DBA genotypes that have increased erythroid output after hG1E-GATA1 treatment are shown, and the size of the bars represents the frequency of that genotype in all DBA patients with an identified gene mutation [S3].

H. Normalized erythroid ratio after treatment of total BM MNCs from DBA patients. On day 6 of erythroid culture, the erythroid maturation ratio was calculated by dividing the percentage of $CD71^+CD235a^+$ cells by the percentage of $CD71^+CD235a^-$ cells and was then normalized to the erythroid maturation ratio of the HMD-GFP treated control. Samples marked in red font are derived from the sample patients as the CD34-selected samples in **Fig. 4G**. Number of markers represents the number of replicates (1 or 3). Mean and S.E.M are shown where appropriate.

I. Quantification of myeloid colonies from DBA patients. 30,000 human bone marrow mononuclear cells from DBA patients or healthy donor controls were plated in methylcellulose. CFU-G, CFU-M, and CFU-GM colonies were counted on day 12. Colonies were imaged and identified using StemVision with manual verification. n = 3-6 independent replicates, mean and S.E.M are shown.

Figure S5. hG1E-GATA1 stimulates erythroid output in DBA samples *in vivo*, related to Figure 5.

A. Human hematopoietic lineages from DBA patient sample BCH-006 after xenotransplantation. Lineages were determined using flow cytometry for the following lineage-defining markers: HSPC, $CD34^+$, T-cell, $CD45^+CD3^+$ B-cell, $CD45^+CD19^+$; myeloid, $CD45^+CD33^+$; megakaryocyte, $CD41a^+$. Each marker represents a recipient mouse with human chimerism >1%. Mean and S.E.M are shown. ns – not significant.

B. Human erythroid committed cells in the bone marrow of recipient mice. Total bone marrow cells were harvested from recipient mice at week 16 after xenotransplantation with treated DBA patient BCH-006 cells, and were analyzed by flow cytometry. Committed erythroid progenitors, defined as having high expression of human CD71, were normalized to the human chimerism in each recipient. Number of individual recipient mice with human chimerism >1% is indicated by the number of markers in each group. Mean and S.E.M. are shown. Two-sided Student *t*-test was used for comparisons. ns – not significant.

C. Increased erythroid maturation *in vivo*. 16 weeks after xenotransplantation with gene therapy treated whole bone marrow MNCs cells from DBA patient BCH-001 or a non-DBA control, bone marrow harvest from recipient mice was performed. The erythroid maturation ratio was calculated by dividing the percentage of $CD235a^+$ cells by the percentage of $CD71^+CD235a^-$ cells and was then normalized to the erythroid maturation ratio from the bone marrow of non-DBA controls. Number of markers represents the number of individual recipient mice per group. Mean and S.E.M. are shown. Two-sided Student *t*-test was used for comparisons. P values are shown, ns – not significant.

D. Cell expansion *in vitro* after xenotransplantation. CD34 selected samples from DBA patient BCH-006 were harvested from xenotransplant recipients after treatment with the indicated vectors as described in **Fig. 5E-G** and were counted by Trypan blue exclusion on day 21. n = 3 independent replicates, mean and S.E.M are shown. Two-sided Student *t*-test was used for comparisons. P value is shown.

E.- F. Erythroid progenitor expansion *in vitro* after xenotransplantation. $CD71^{+}$ cells from BCH-006 were harvested from xenotransplant recipients after treatment with the indicated vectors as described in **Fig. 5E-G** and were counted by Trypan blue exclusion on the indicated days (**E**). Two-sided Student *t*-test was used for comparisons. * P = 0.005, ** P = 0.0002. Absent error bars are obscured by the size of the markers. (**F**) Total erythroid number was determined by multiplying total cell number by percentage of CD235a cells on day 21 as determined by flow cytometry n = 3 independent replicates, mean and S.E.M are shown. Two-sided Student *t*-test was used for comparisons. P values are shown.

Figure S6. Transcriptional effects of hG1E-GATA1 therapy, related to Figure 6.

A. UMAP projection of the expression of erythroid and other lineage-defining markers in hematopoietic cells from DBA patients BCH-002 and BCH-008 on day 10 of erythroid culture. Hematopoietic lineages of the cellular clusters are as labeled in **Fig. 6A**.

B.- C. UMAP projection of cells with an erythroid signature from DBA patients BCH-002 and BCH-008 further colored by Seurat clusters defined by stage of differentiation (**B.**) and sample identity (**C.**). GFP: HMD-GFP, hG1E: hG1E-GATA1. After selecting erythroid cells, a new dimensionality reduction was performed by PCA, and the top 30 PCs were used to determine the UMAP projection of erythroid subset.

D. Expression of selected erythroid genes and hG1E-GATA1 transgene across pseudotime and colored by Seurat clusters from **Fig. S6B**.

E. Violin plot of pseudotime value for each defined Seurat cluster of erythroid cells, as shown in **Fig. S6B**.

F. Discrimination of hG1E-GATA1 transgene expression. Codon optimized GATA1 expression from the hG1E-GATA1 vector is shown per sample and is detected only in hG1E-GATA1 treated cells.

G. Gene set enrichment analysis (GSEA) plot showing depletion of Hallmark p53 pathway genes following hG1E-GATA1 treatment in individual DBA patients BCH-002 and BCH-008. Normalized enrichment score (NES) and p-value are shown. The Kolmogorov Smirnov (K-S) test was used to determine the significance of GSEA.

Figure S7. Safe genomic integration of hG1E-GATA1, related to Figure 7.

A. Cumulative frequencies of integration sites of hG1E-GATA1 following transduction in bulk HSPCs and in erythroid cells on day 18 of *in vitro* culture. RefSeq names of the genes closest to integration sites are displayed.

B. Comparison of integration sites of hG1E-GATA1 in erythroid cells on day 18 of *in vitro* culture. Integration sites were compared to the integration profile of a control lentivirus and are organized by chromosome location along the x-axis. Relative gene targeting score is shown on the y-axis. No integration sites are significantly enriched in the hG1E-GATA1 treated samples (dotted lines).

Supplemental References

S1. Schulz, V.P., Yan, H., Lezon-Geyda, K., An, X., Hale, J., Hillyer, C.D., Mohandas, N., and Gallagher, P.G. (2019). A unique epigenomic landscape defines human erythropoiesis. Cell Rep. *28*, 2996-3009.e2997. 10.1016/j.celrep.2019.08.020.

S2. Su, M.Y., Steiner, L.A., Bogardus, H., Mishra, T., Schulz, V.P., Hardison, R.C., and Gallagher, P.G. (2013). Identification of biologically relevant enhancers in human erythroid cells. J. Biol. Chem. *288*, 8433-8444. 10.1074/jbc.m112.413260.

S3. Ulirsch, J.C., Verboon, J.M., Kazerounian, S., Guo, M.H., Yuan, D., Ludwig, L.S., Handsaker, R.E., Abdulhay, N.J., Fiorini, C., Genovese, G., et al. (2018). The genetic landscape of Diamond-Blackfan anemia. Am. J. Hum. Genet. *103*, 930-947. 10.1016/j.ajhg.2018.10.027.
